# Medical image analysis using deep learning algorithms

**DOI:** 10.3389/fpubh.2023.1273253

**Published:** 2023-11-07

**Authors:** Mengfang Li, Yuanyuan Jiang, Yanzhou Zhang, Haisheng Zhu

**Affiliations:** ^1^The First Affiliated Hospital of Wenzhou Medical University, Wenzhou, China; ^2^Department of Cardiovascular Medicine, The First Affiliated Hospital of Zhengzhou University, Zhengzhou, China; ^3^Department of Cardiovascular Medicine, Wencheng People’s Hospital, Wencheng, China

**Keywords:** deep learning, machine learning, medical images, image analysis, convolutional neural networks

## Abstract

In the field of medical image analysis within deep learning (DL), the importance of employing advanced DL techniques cannot be overstated. DL has achieved impressive results in various areas, making it particularly noteworthy for medical image analysis in healthcare. The integration of DL with medical image analysis enables real-time analysis of vast and intricate datasets, yielding insights that significantly enhance healthcare outcomes and operational efficiency in the industry. This extensive review of existing literature conducts a thorough examination of the most recent deep learning (DL) approaches designed to address the difficulties faced in medical healthcare, particularly focusing on the use of deep learning algorithms in medical image analysis. Falling all the investigated papers into five different categories in terms of their techniques, we have assessed them according to some critical parameters. Through a systematic categorization of state-of-the-art DL techniques, such as Convolutional Neural Networks (CNNs), Recurrent Neural Networks (RNNs), Generative Adversarial Networks (GANs), Long Short-term Memory (LSTM) models, and hybrid models, this study explores their underlying principles, advantages, limitations, methodologies, simulation environments, and datasets. Based on our results, Python was the most frequent programming language used for implementing the proposed methods in the investigated papers. Notably, the majority of the scrutinized papers were published in 2021, underscoring the contemporaneous nature of the research. Moreover, this review accentuates the forefront advancements in DL techniques and their practical applications within the realm of medical image analysis, while simultaneously addressing the challenges that hinder the widespread implementation of DL in image analysis within the medical healthcare domains. These discerned insights serve as compelling impetuses for future studies aimed at the progressive advancement of image analysis in medical healthcare research. The evaluation metrics employed across the reviewed articles encompass a broad spectrum of features, encompassing accuracy, sensitivity, specificity, F-score, robustness, computational complexity, and generalizability.

## 1. Introduction

Deep learning is a branch of machine learning that employs artificial neural networks comprising multiple layers to acquire and discern intricate patterns from extensive datasets (1, 2). It has brought about a revolution in various domains, including computer vision, natural language processing, and speech recognition, among other areas (3). One of the primary advantages of deep learning is its capacity to automatically learn features from raw data, thereby eliminating the necessity for manual feature engineering (4). This makes it especially powerful in domains with large, complex datasets, where traditional machine learning methods may struggle to capture the underlying patterns (5). Deep learning has also facilitated significant advancements in various tasks, including but not limited to image and speech recognition, comprehension of natural language, and the development of autonomous driving capabilities (6). For instance, deep learning has enabled the creation of exceptionally precise computer vision systems capable of identifying objects in images and videos with unparalleled precision. Likewise, deep learning has brought about substantial enhancements in natural language processing, leading to the development of models capable of comprehending and generating language that resembles human-like expression (7). Overall, deep learning has opened up new opportunities for solving complex problems and has the potential to transform many industries, including healthcare, finance, transportation, and more.

Medical image analysis is a field of study that involves the processing, interpretation, and analysis of medical images (8). The emergence of deep learning algorithms has prompted a notable transformation in the field of medical image analysis, as they have increasingly been employed to enhance the diagnosis, treatment, and monitoring of diverse medical conditions in recent years (9). Deep learning, as a branch of machine learning, encompasses the training of algorithms to acquire knowledge from vast quantities of data. When applied to medical image analysis, deep learning algorithms possess the capability to automatically identify and categorize anomalies in various medical images, including X-rays, MRI scans, CT scans, and ultrasound images (10). These algorithms can undergo training using extensive datasets consisting of annotated medical images, where each image is accompanied by labels indicating the corresponding medical condition or abnormality (11). Once trained, the algorithm can analyze new medical images and provide diagnostic insights to healthcare professionals. The application of deep learning algorithms in medical image analysis has exhibited promising outcomes, as evidenced by studies showcasing high levels of accuracy in detecting and diagnosing a wide range of medical conditions (12). This has led to the development of various commercial and open-source software tools that leverage deep learning algorithms for medical image analysis (13). Overall, the utilization of deep learning algorithms in medical image analysis has the capability to bring about substantial enhancements in healthcare results and transform the utilization of medical imaging in diagnosis and treatment.

Medical image processing is an area of research that encompasses the creation and application of algorithms and methods to analyze and decipher medical images (14). The primary objective of medical image processing is to extract meaningful information from medical images to aid in diagnosis, treatment planning, and therapeutic interventions (15). Medical image processing involves various tasks such as image segmentation, image registration, feature extraction, classification, and visualization. The primary aim of medical image processing is to extract pertinent information from medical images, facilitating the tasks of diagnosis, treatment planning, and therapeutic interventions. Each modality has its unique strengths and limitations, and the images produced by different modalities may require specific processing techniques to extract useful information (16). Medical image processing techniques have revolutionized the field of medicine by providing a non-invasive means to visualize and analyze the internal structures and functions of the body. It has enabled early detection and diagnosis of diseases, accurate treatment planning, and monitoring of treatment response. The use of medical image processing has significantly improved patient outcomes, reduced treatment costs, and enhanced the quality of care provided to patients. Visual depictions of CNNs in the context of medical image analysis using DL algorithms portray a layered architecture, where initial layers capture rudimentary features like edges and textures, while subsequent layers progressively discern more intricate and abstract characteristics, allowing the network to autonomously extract pertinent information from medical images for tasks like detection, segmentation, and classification. Additionally, the visual representations of RNNs in medical image analysis involving DL algorithms illustrate a network structure adept at grasping temporal relationships and sequential patterns within images, rendering them well-suited for tasks such as video analysis or the processing of time-series medical image data. Furthermore, visual representations of GANs in medical image analysis employing DL algorithms exemplify a dual-network framework: one network, the generator, fabricates synthetic medical images, while the other, the discriminator, assesses their authenticity, facilitating the generation of lifelike images closely resembling actual medical data. Moreover, visual depictions of LSTM networks in medical image analysis with DL algorithms delineate a specialized form of recurrent neural network proficient in processing sequential medical image data by preserving long-term dependencies and learning temporal patterns crucial for tasks like video analysis and time-series image processing. Finally, visual representations of hybrid methods in medical image analysis using DL algorithms portray a combination of diverse neural network architectures, often integrating CNNs with RNNs or other specialized modules, enabling the model to harness both spatial and temporal information for a comprehensive analysis of medical images.

Case studies and real-world examples provide tangible evidence of the effectiveness and applicability of DL algorithms in various medical image analysis tasks. They underscore the potential of this technology to revolutionize healthcare by improving diagnostic accuracy, reducing manual labor, and enabling earlier interventions for patients. Here are several examples of case studies and real-worlds applications:

1. Skin cancer detection
  Case Study: In Vijayalakshmi (17), a DL algorithm was trained to identify skin cancer from images of skin lesions. The algorithm demonstrated accuracy comparable to that of dermatologists, highlighting its potential as a tool for early skin cancer detection.
2. Diabetic retinopathy screening
  Case Study: also, De Fauw et al. (18) in Moorfields Eye Hospital, developed a DL system capable of identifying diabetic retinopathy from retinal images. The system was trained on a dataset of over 128,000 images and achieved a level of accuracy comparable to expert ophthalmologists.

1. Tumor segmentation in MRI
  Case Study: A study conducted by Guo et al. (8) at Massachusetts General Hospital utilized DL techniques to automate the segmentation of brain tumors from MRI scans. The algorithm significantly reduced the time required for tumor delineation, enabling quicker treatment planning for patients.
2. Chest X-ray analysis for tuberculosis detection
  Case Study: The National Institutes of Health (NIH) released a dataset of chest X-ray images for the detection of tuberculosis. Researchers have successfully applied deep learning algorithms to this dataset, achieving high accuracy in identifying TB-related abnormalities.
3. Automated bone fracture detection
  Case Study: Meena and Roy (19) at Stanford University developed a deep learning model capable of detecting bone fractures in X-ray images. The model demonstrated high accuracy and outperformed traditional rule-based systems in fracture detection.

Within the realm of medical image analysis utilizing DL algorithms, ML algorithms are extensively utilized for precise and efficient segmentation tasks. DL approaches, especially convolutional neural networks (CNNs) and recurrent neural networks (RNNs), have demonstrated exceptional proficiency in capturing and leveraging spatial dependencies and symmetrical properties inherent in medical images. These algorithms enable the analyzing medical image of symmetric structures, such as organs or limbs, by leveraging their inherent symmetrical patterns. The utilization of DL mechanisms in medical image analysis encompasses various practical approaches, including generative adversarial networks (GANs), hybrid models, and combinations of CNNs and RNNs. The objective of this research is to offer a thorough examination of the uses of DL techniques in the domain of deep symmetry-based image analysis within medical healthcare, providing a comprehensive overview. By conducting an in-depth systematic literature review (SLR), analyzing multiple studies, and exploring the properties, advantages, limitations, datasets, and simulation environments associated with different DL mechanisms, this study enhances comprehension regarding the present state and future pathways for advancing and refining deep symmetry-based image analysis methodologies in the field of medical healthcare. The article is structured in the following manner: The key principles and terminology of ML/DL in medical image analysis are covered in the first part, followed by an investigation of relevant papers in part 3. Part 4 discusses the studied mechanisms and tools for paper selection, while part 5 illustrates the classification that was selected. Section 6 presents the results and comparisons, and the remaining concerns and conclusion are explored in the last section.

## 2. Fundamental concepts and terminology

The concepts and terms related to medical image analysis using DL algorithms that are covered in this section are essential for understanding the underlying principles and techniques used in medical image analysis.

### 2.1. The role of image analysis in medical healthcare

The utilization of deep learning algorithms for image analysis has brought about a revolution in medical healthcare by facilitating advanced and automated analysis of medical images (20). Deep learning methods, including Convolutional Neural Networks (CNNs), have showcased outstanding proficiency in tasks like image segmentation, feature extraction, and classification, exhibiting remarkable performance (21). By leveraging large amounts of annotated data, deep learning models can learn intricate patterns and relationships within medical images, facilitating accurate detection, localization, and diagnosis of diseases and abnormalities. Deep learning-based image analysis allows for faster and more precise interpretation of medical images, leading to improved patient outcomes, personalized treatment planning, and efficient healthcare workflows (22). Furthermore, these algorithms have the potential to assist in early disease detection, assist radiologists in decision-making, and enhance medical research through the analysis of large-scale image datasets. Overall, deep learning-based image analysis is transforming medical healthcare by providing powerful tools for image interpretation, augmenting the capabilities of healthcare professionals, and enhancing patient care (23).

### 2.2. Medical image analysis application

The utilization of deep learning algorithms in medical image analysis has discovered numerous applications within the healthcare sector. Deep learning techniques, notably Convolutional Neural Networks (CNNs), have been widely employed for tasks encompassing image segmentation, object detection, disease classification, and image reconstruction (24). In medical image analysis, these algorithms can assist in the detection and diagnosis of various conditions, such as tumors, lesions, anatomical abnormalities, and pathological changes. They can also aid in the evaluation of disease progression, treatment response, and prognosis. Deep learning models can automatically extract meaningful features from medical images, enabling efficient and accurate interpretation (25). The application of this technology holds promise for elevating clinical decision-making, ameliorating patient outcomes, and optimizing resource allocation in healthcare settings. Moreover, deep learning algorithms can be employed for data augmentation, image registration, and multimodal fusion, facilitating a comprehensive and integrated analysis of medical images obtained from various modalities. With continuous advancements in deep learning algorithms, medical image analysis is witnessing significant progress, opening up new possibilities for precision medicine, personalized treatment planning, and advanced healthcare solutions (26).

### 2.3. Various aspects of medical image analysis for the healthcare section

Medical image analysis encompasses various crucial aspects in the healthcare sector, enabling in-depth examination and diagnosis based on medical imaging data (27). Image preprocessing constitutes a crucial element, encompassing techniques like noise reduction, image enhancement, and normalization, aimed at enhancing the quality and uniformity of the images. Another essential aspect is image registration, which aligns multiple images of the same patient or acquired through different imaging modalities, enabling precise comparison and fusion of information (28). Feature extraction is another crucial step, where relevant characteristics and patterns are extracted from the images, aiding in the detection and classification of abnormalities or specific anatomical structures. Segmentation plays a vital role in delineating regions of interest, enabling precise localization and measurement of anatomical structures, tumors, or lesions (29). Finally, classification and recognition techniques are applied to differentiate normal and abnormal regions, aiding in disease diagnosis and treatment planning. Deep learning algorithms, notably Convolutional Neural Networks (CNNs), have exhibited extraordinary achievements in diverse facets of medical image analysis by acquiring complex patterns and representations from extensive datasets of medical imaging (30). However, challenges such as data variability, interpretability, and generalization across different patient populations and imaging modalities need to be addressed to ensure reliable and effective medical image analysis in healthcare applications.

## 3. Relevant reviews

We are going to look into some recent research on medical image analysis using DL algorithms in this part. The initial purpose is to properly make a distinction between the current study’s significant results in comparison with what is discussed in this paper. Due to advancements in AI technology, there is a growing adoption of AI mechanisms in medical image analysis. Simultaneously, academia has shown a heightened interest in addressing challenges related to medical image analysis. Furthermore, medical image analysis is a hierarchical network management framework modeled to direct analysis availability to aim medical healthcare. In this regard, Gupta and Katarya (31) provided a comprehensive review of the literature on social media-based surveillance systems for healthcare using machine learning. The authors analyzed 50 studies published between 2011 and 2021, covering a wide range of topics related to social media monitoring for healthcare, including disease outbreaks, adverse drug reactions, mental health, and vaccine hesitancy. The review highlighted the potential of machine learning algorithms for analyzing vast amounts of social media data and identifying relevant health-related information. The authors also identified several challenges associated with the use of social media data, such as data quality and privacy concerns, and discuss potential solutions to address these challenges. The authors noted that social media-based surveillance systems can complement traditional surveillance methods by providing real-time data on health-related events and trends. They also suggested that machine learning algorithms can improve the accuracy and efficiency of social media monitoring by automatically filtering out irrelevant information and identifying patterns and trends in the data. The review highlighted the importance of data pre-processing and feature selection in developing effective machine learning models for social media analysis.

As well, Kourou et al. (32) reviewed machine learning (ML) applications for cancer prognosis and prediction. The authors started by describing the challenges of cancer treatment, highlighting the importance of personalized medicine and the role of ML algorithms in enabling it. The paper then provided an overview of different types of ML algorithms, including supervised and unsupervised learning, and discussed their potential applications in cancer prognosis and prediction. The authors presented examples of studies that have used ML algorithms for diagnosis, treatment response prediction, and survival prediction across different types of cancer. They also discussed the use of multiple data sources for ML algorithms, such as genetic data, imaging data, and clinical data. The paper concluded by addressing the challenges and limitations encountered in using ML algorithms for cancer prognosis and prediction, which include concerns regarding data quality, overfitting, and interpretability. The authors proposed that ML algorithms hold significant potential for enhancing cancer treatment outcomes. However, they emphasized the necessity for further research to optimize their application and tackle the associated challenges in this domain.

Moreover, Razzak et al. (33) provided a comprehensive overview of the use of deep learning in medical image processing. The authors deliberated on the potential of deep learning algorithms in diverse medical imaging tasks, encompassing image classification, segmentation, registration, and synthesis. They emphasized the challenges encountered when employing deep learning, such as the requirement for extensive annotated datasets, interpretability of deep models, and computational demands. Additionally, the paper delved into prospective avenues in the field, including the integration of multi-modal data, transfer learning, and the utilization of generative models. In summary, the paper offered valuable perspectives on the present status, challenges, and potential advancements of deep learning in the domain of medical image processing.

In addition, Litjens et al. (34) provided a comprehensive survey of the applications of deep learning in medical image analysis. A thorough introduction of the deep learning approaches used in each of these areas is provided by the authors as they look at a variety of tasks in medical imaging, including picture classification, segmentation, detection, registration, and creation. Additionally, they look at the difficulties and restrictions of using deep learning algorithms for medical image analysis, such as the need for sizable datasets with annotations and the interpretability of deep models. The growth of explainable and interpretable deep learning models is highlighted in the paper’s conclusion along with other potential future possibilities in the area, such as the integration of multimodal data. In summary, this survey serves as a valuable resource for researchers and practitioners, offering insights into the current state and future prospects of deep learning in the context of medical image analysis.

Additionally, Bzdok and Ioannidis (35) discussed the importance of exploration, inference, and prediction in the fields of neuroscience and biomedicine. The author highlighted the importance of integrating diverse data types, such as neuroimaging, genetics, and behavioral data, in order to achieve a comprehensive comprehension of intricate systems. Bzdok also delved into the role of machine learning in facilitating the identification of patterns and making predictions based on extensive datasets. The author provided an account of several specific applications of machine learning in neuroscience and biomedicine, including forecasting disease progression and treatment response, analyzing brain connectivity networks, and identifying biomarkers for disease diagnosis. The paper concluded by discussing the challenges and limitations encountered when employing machine learning in these domains, while emphasizing the essentiality of carefully considering the ethical and social implications of these technologies. Moreover, the paper underscored the potential of machine learning to transform our understanding of complex biological systems and enhance medical outcomes. Table 1 depicts summary of related works.

**Table 1:** Summary of related works.

Author	Main idea	Advantage	Disadvantage
Gupta and Katarya (31)	Providing a comprehensive review of the literature on social media-based surveillance systems for healthcare using machine learning	Aiming to study the latest trends in surveillance systems using social media and machine learning algorithms in the area of public health	Poor schematic comparison between investigated methods
Kourou, et al. (32)	Reviewing ML applications for cancer prognosis and prediction	Addressing the challenges and considerations in utilizing machine learning in the field of oncology, such as data availability, feature selection, and model interpretability	Limited data availability Lack of clinical validation Limited interpretability
Razzak, et al. (33)	Providing a comprehensive overview of the use of deep learning in medical image processing	Comprehensive overview Identification of challenges Future directions Integration of interdisciplinary knowledge	Limited scope Outdated information Lack of empirical validation Bias or limited perspectives
Litjens, et al. (34)	Providing a comprehensive survey of the applications of deep learning in medical image analysis	Comprehensive overview Inclusion of state-of-the-art methods Extensive citation and references Organization and clarity	Limited scope and coverage Lack of recent developments Bias in selection and representation Insufficient critical analysis Length and level of detail
Bzdok and Ioannidis (35)	Discussing the importance of exploration, inference, and prediction in the fields of neuroscience and biomedicine	Comprehensive coverage Integration of diverse perspectives Timely and up-to-date information Critical analysis and insights Clear and accessible writing style	Limited scope Lack of empirical evidence Potential bias Lack of recent updates Limited generalizability
Our work	Introducing a new taxonomy of DL methods in medical image analysis	Comprehensive investigation on the topic from different aspects	Lack of investigation on non-English papers

## 4. Methodology of research

We thoroughly examined pertinent documents that partially explored the utilization of DL methods in medical image analysis. By utilizing the Systematic Literature Review (SLR) methodology, this section comprehensively encompasses the field of medical image analysis. The SLR technique encompasses a thorough evaluation of all research conducted on a significant topic. This section concludes with an extensive investigation of ML techniques in the realm of medical image analysis. Furthermore, the reliability of the research selection methods is scrutinized. In the subsequent subsections, we have provided supplementary information concerning research techniques, encompassing the selection metrics and research inquiries.

### 4.1. Formalization of question

The primary aims of the research are to identify, assess, and differentiate all key papers within the realm of using DL methods medical image analysis. A systematic literature review (SLR) can be utilized to scrutinize the constituents and characteristics of methods for accomplishing the aforementioned objectives. Furthermore, an SLR facilitates the acquisition of profound comprehension of the pivotal challenges and difficulties in this domain. The following paragraph outlines several research inquiries:

Research Question 1: In what manners can DL techniques in the field of medical image analysis be categorized? The answer to this question can be found in Part 5.

Research Question 2: What types of techniques do scholars employ to execute their investigation? Parts 5.1 to 5.7 elucidate this query.

Research Question 3: Which parameters attracted the most attention in the papers? What are the most popular DL applications utilized in medical image analysis? The answer to this question is included in Part 6.

Research Question 4: What unexplored prospects exist in this area? Part 7 proffers the answer to this question.

### 4.2. The procedure of paper exploration

**Figure 1:**
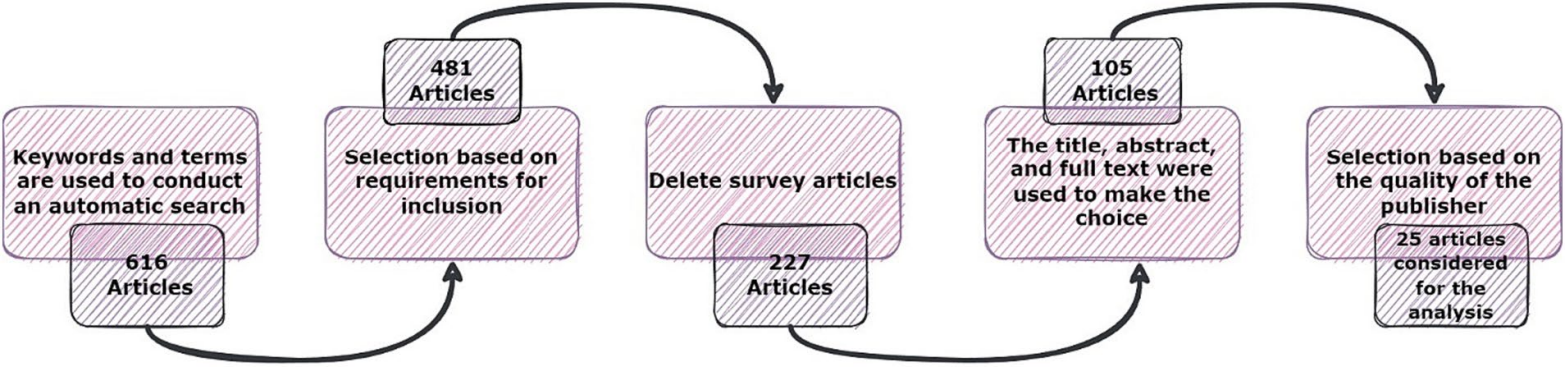
The phases of the article searching and selection process.

**Figure 2:**
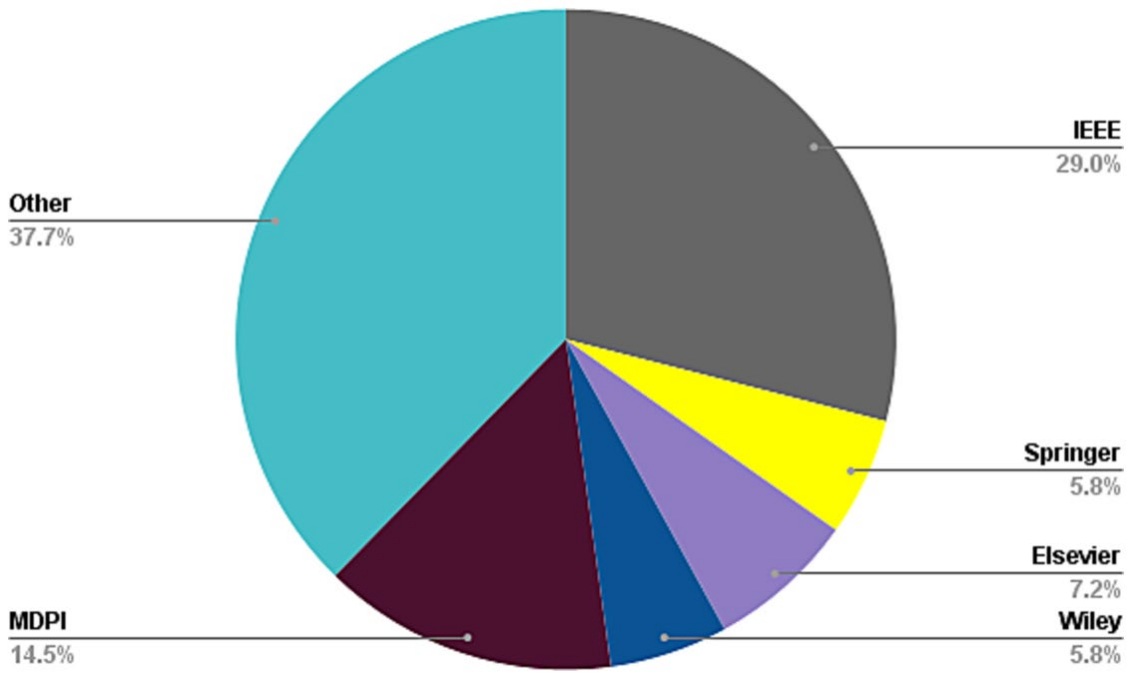
Frequency of publications of studied paper in first stage of paper selection.

**Figure 3:**
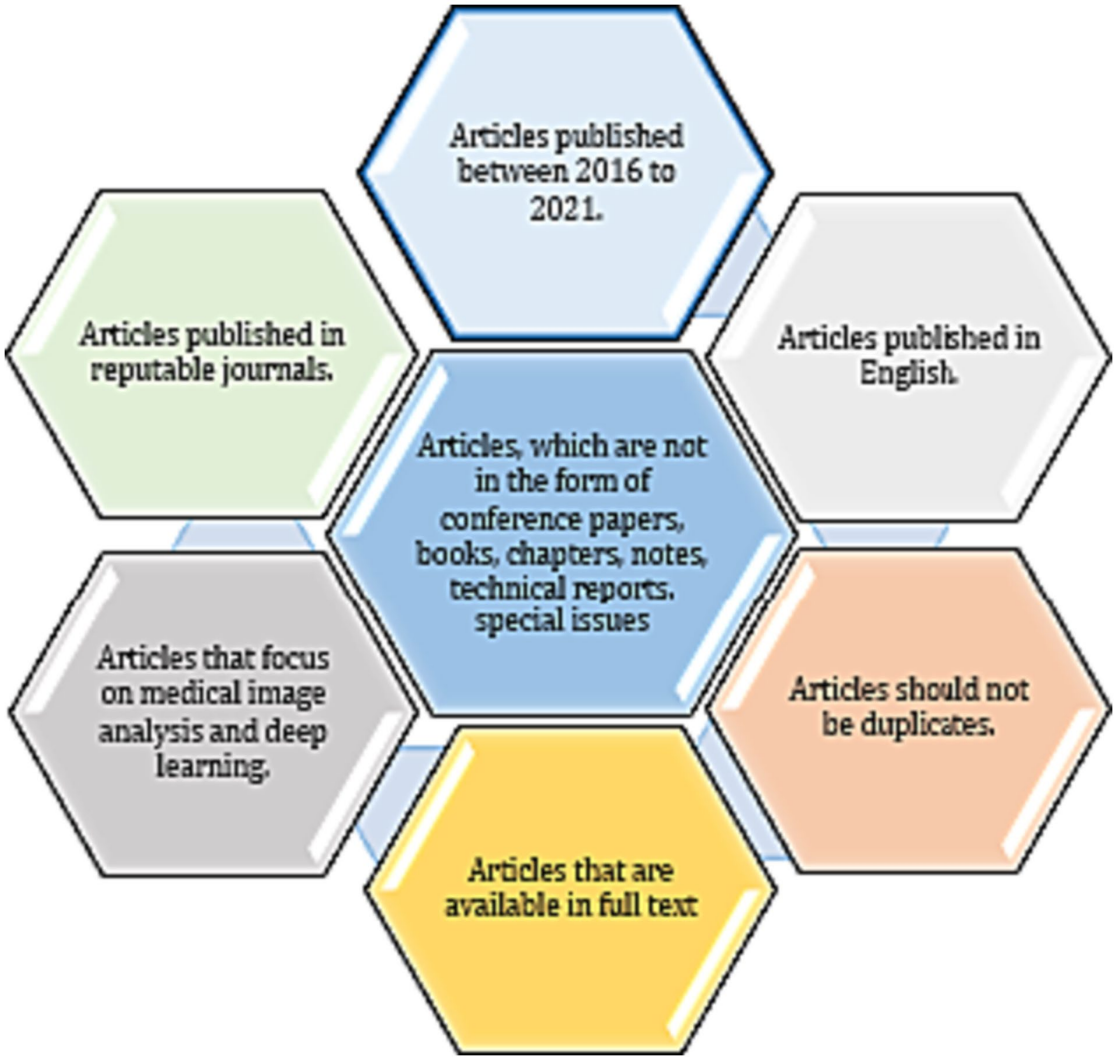
Criteria for inclusion in the paper selection process.

**Figure 4:**
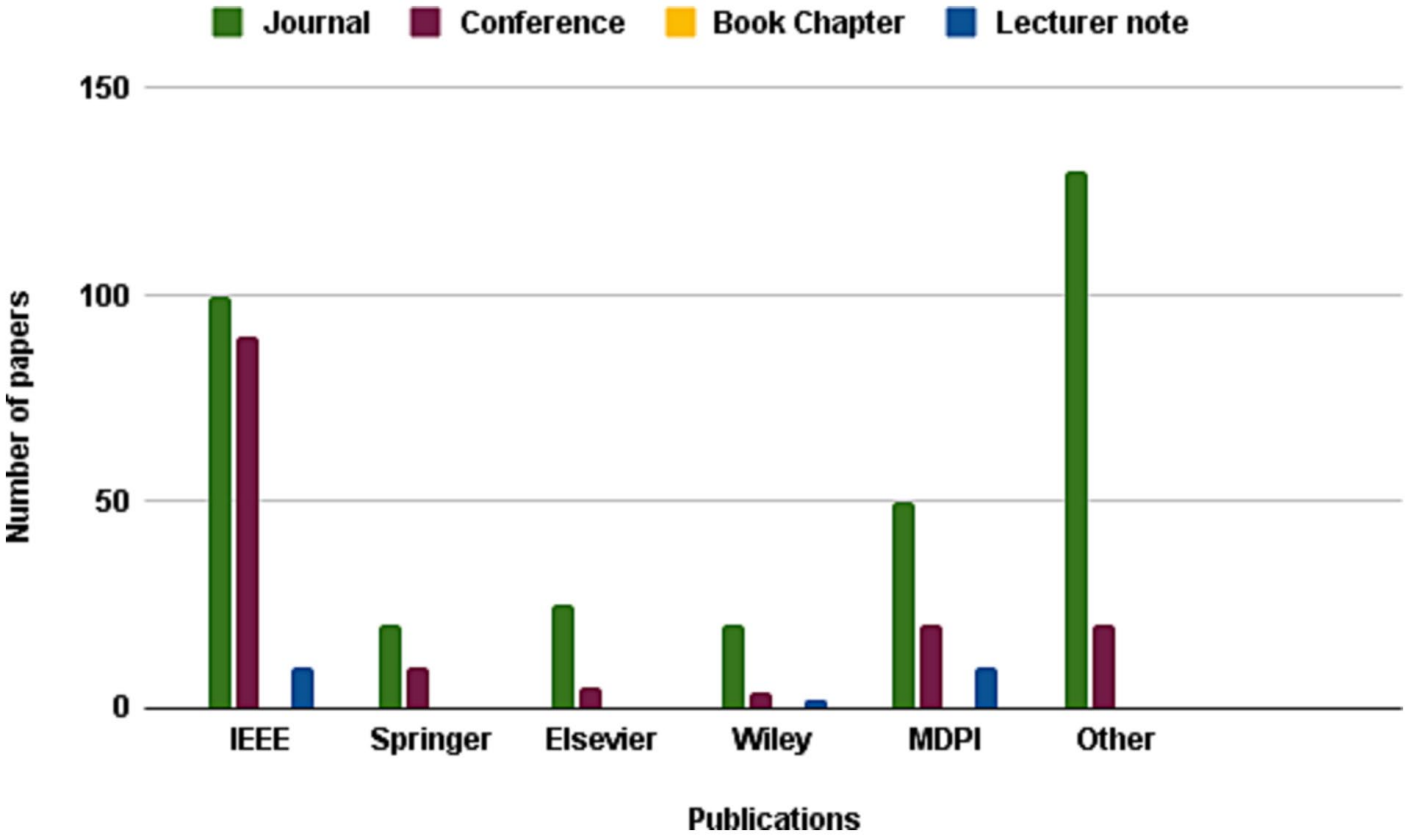
Frequency of publications of studied paper in second stage of paper selection.

The present investigation’s pursuit and selection methodologies are classified into four distinct phases, as depicted in Figure 1. In the initial phase, a comprehensive list of keywords and phrases was utilized to scour various sources, as demonstrated in Table 2. An electronic database was employed to retrieve relevant documents, including Chapters, Journals, technical studies, conference papers, notes, and special issues, resulting in a total of 616 papers as is shown if Figure 2. These papers were then subjected to an exhaustive analysis based on a set of predetermined standards, and only those meeting the stipulated criteria, illustrated in Figure 3, were selected for further evaluation. The distribution of publishers in this initial phase is shown in Figure 4, and the number of articles left after the first phase was 481.

**Table 2:** Keywords and search criteria.

S#	Keywords and search criteria	S#	Keywords and search criteria
S1	“DL” and “Medical”	S6	“AI” and “Healthcare”
S2	“ML” and “Healthcare”	S7	“Healthcare” and “DL algorithms”
S3	“DL” and “Image Analysis”	S8	“DL methods” and “Medical Images”
S4	“ML” and “Medical Healthcare”	S9	“Image Analysis” and “Medical Healthcare”
S5	“AI” and “Medical Healthcare”	S10	“AI methods” and “Medical Images”

**Figure 5:**
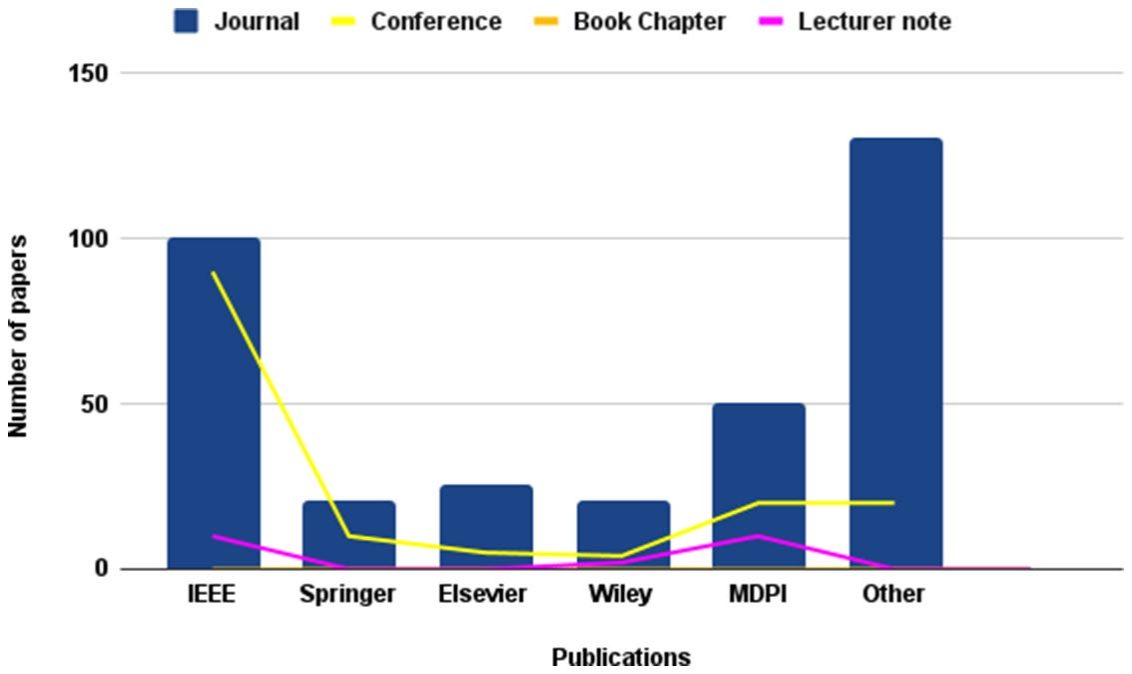
Frequency of publications of studied paper in third stage of paper selection.

**Figure 6:**
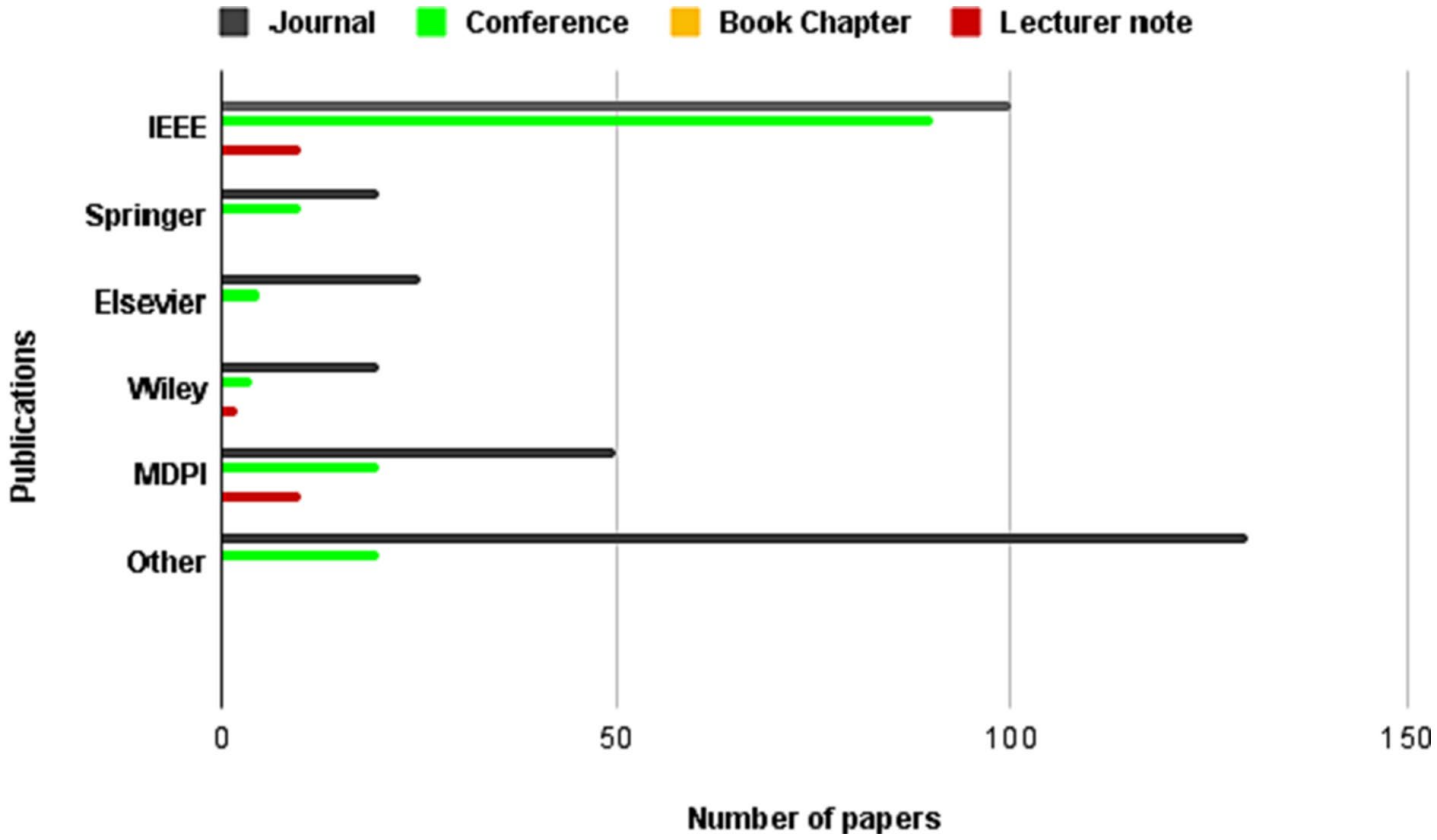
Frequency of publications of studied paper in forth stage of paper selection.

In the subsequent phase, a thorough review of the selected papers’ titles and abstracts was conducted, focusing on the papers’ discussion, methodology, analysis, and conclusion to ensure their relevance to the study. As demonstrated in Figure 5, only 227 papers were retained after this step and 105 papers were further.

chosen for a more comprehensive review, as illustrated in Figure 6, with the ultimate aim of selecting papers that adhered to the study’s predetermined metrics. Finally, after careful consideration, 25 articles were hand-picked to investigate other publications.

## 5. ML/DL techniques for medical image analysis

**Figure 7:**
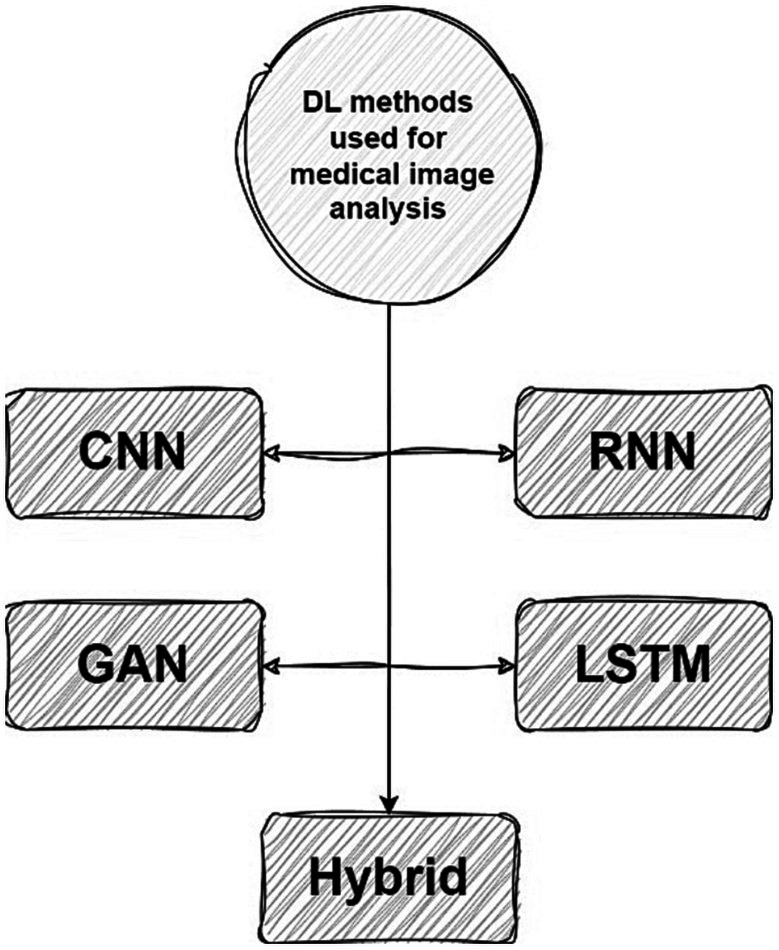
The proposed taxonomy of Bioinformatics.

In this section, we delve into the implementation of DL methods in the medical healthcare image analysis field. A total of 25 articles satisfying our selection criteria will be presented herein. Initially, we categorize the techniques into 5 primary groups comprising CNNs, RNNs, GANs, LSTMs, and hybrid methodologies encompassing diverse methods. The proposed taxonomy of DL-associated medical image analysis in medical healthcare is depicted in Figure 7.

### 5.1. Convolutional neural network techniques for medical image analysis

When using deep learning approaches for medical image processing, convolutional neural networks (CNNs) play a significant role. They perform well in tasks like object localization, segmentation, and classification due to their capacity to automatically extract pertinent characteristics from intricate medical pictures. CNNs are able to accurately identify anomalies, diagnose tumors, and segment organs in medical pictures by capturing complex patterns and structures. Important characteristics may be learnt at various levels by utilizing the hierarchical structure of CNNs, which improves analysis and diagnosis. Employing CNNs in medical image analysis has notably improved the precision, effectiveness, and automation of diagnostic procedures, ultimately leading to advantageous patient care and treatment results.

In this regard, Singh et al. (36) highlighted the role of artificial intelligence (AI) and machine learning (ML) techniques in advancing biomedical material design and predicting their toxicity. The authors emphasized the need for efficient and safe materials for medical applications and how computational methods can aid in this process. The paper explored diverse categories of AI and ML algorithms, including random forests, decision trees, and support vector machines, which can be employed for predicting toxicity. The authors provided a case study wherein they utilized a random forest algorithm to predict the toxicity of carbon nanotubes. They also highlighted the importance of data quality and quantity for accurate predictions, as well as the need for interpretability and transparency of AI/ML models. The paper concluded by discussing future research directions in this area, including the integration of multi-omics data, network analysis, and deep learning techniques. This paper demonstrated the potential of AI/ML in advancing biomedical material design and reducing the need for animal testing.

Also, Jena et al. (37) investigated the impact of parameters on the performance of deep learning models for the classification of diabetic retinopathy (DR) in a smart healthcare system. Using retinal fundus pictures, the scientists developed a convolutional neural network (CNN) architecture with two branches to categorize diabetic retinopathy (DR). A branch for feature extraction and another for classification are both included in the suggested model. A pre-trained model is used in the feature extraction branch to extract pertinent characteristics from the input picture, and the classification branch uses these features to predict the severity of DR. The learning rate, number of epochs, batch size, and optimizer were among the variables that the authors experimented with in order to evaluate the model’s performance. The outcomes showed that the suggested model, when using the ideal parameter configuration, had an accuracy of 98.12%. The authors also suggested a secure IoT-based blockchain-based smart healthcare system for processing and storing medical data. The proposed system could be used for the early diagnosis and treatment of DR, thereby improving patient outcomes.

As well, Thilagam et al. (38) presented a secure Internet of Things (IoT) healthcare architecture with a deep learning-based access control system. The proposed system is designed to ensure that only authorized personnel can access the sensitive medical information stored in IoT devices. The authors used deep learning algorithms to develop a robust access control system that can identify and authenticate users with high accuracy. The system also included an encryption layer to ensure that all data transmitted between devices is secure. The authors assessed the proposed architecture through a prototype implementation, which revealed that the system can securely access medical data in real-time. Additionally, the authors conducted a comparison with existing solutions and demonstrated that their approach outperforms others in terms of accuracy, security, and scalability. The paper underscored the potential of employing deep learning algorithms in healthcare systems to enhance security and privacy, while facilitating real-time access to medical data.

Besides, Ismail et al. (39) proposed a CNN-based model for analyzing regular health factors in an IoMT (Internet-of-Medical-Things) environment. The model extracted feature from multiple health data sources, such as blood pressure, pulse rate, and body temperature, using CNN-based algorithms, which are then used to predict the risk of health issues. The proposed model is capable of classifying health data into five categories: normal, pre-hypertension, hypertension, pre-diabetes, and diabetes. The authors utilized a real-world dataset comprising health data from 50 individuals to train and evaluate the model. The findings indicated that the proposed model exhibited a remarkable level of accuracy and surpassed existing machine learning models in terms of both predictive accuracy and computational complexity. The authors expressed their confidence that the proposed model could contribute to the advancement of health monitoring systems, offering real-time monitoring and personalized interventions, thereby preventing health issues and enhancing patient outcomes.

And, More et al. (40) proposed a security-assured CNN-based model for the reconstruction of medical images on the Internet of Healthcare Things (IoHT) with the goal of ensuring the privacy and security of medical data. The proposed framework comprises two main components: a deep learning-based image reconstruction model and a security-enhanced encryption model. The image reconstruction model relies on a convolutional neural network (CNN) to accurately reconstruct original medical images from compressed versions. To safeguard the transmitted images, the encryption model employs a hybrid encryption scheme that combines symmetric and asymmetric techniques. Through evaluation using a widely recognized medical imaging dataset, the results demonstrated the model’s remarkable reconstruction accuracy and effective security performance. This study underscores the potential of leveraging deep learning models in healthcare, particularly within medical image processing, while emphasizing the crucial need for ensuring the security and privacy of medical data. Table 3 discusses the CNN methods used in medical image analysis and their properties.

**Table 3:** The methods, properties, and features of CNN-medical image analysis mechanisms.

Author	Main idea	Advantage	Disadvantage	Simulation environment	Datasets
Singh, et al. (36)	Presenting a case study where they employed a random forest algorithm for toxicity prediction of carbon nanotubes	High integrity Cost-effective	Reliance on high-quality and representative training data Potential biases within the dataset interpretability and explainability of the ML model’s predictions Need for validation and verification of the model’s accuracy and reliability	Python	27 observations
Jena, et al. (37)	Proposing a 2-branch convolutional neural network (CNN) architecture to classify DR in retinal fundus images	High specificity High sensitivity High F-score High AUC	Need for large amounts of labeled training data Computational resource requirements Interpretability of the model’s decision-making process	Python	Fundus images of 102 diabetic patients
Thilagam, et al. (38)	Presenting a secure Internet of Things (IoT) healthcare architecture with a deep learning-based access control system	High security High robustness High privacy High accuracy	Computational complexity Resource requirements associated with deep learning algorithms Requiring a large amount of labeled data for training Limited generalizability	Python	100 participants performing 10 different gestures and activities over a duration of 60 s each
Ismail, et al. (39)	Proposing a CNN-based model for analyzing regular health factors in an IoMT (Internet-of-Medical-Things) environment	High accuracy	Reliance on CNNs as the sole modeling technique	Python	Real-time health examinations of 10,806 citizens
More, et al. (40)	Proposing a security-assured CNN-based model for the reconstruction of medical images on the Internet of Healthcare Things (IoHT)	High accuracy High security High sensitivity High reliability	Computational complexity Resource requirements Limited storage capacity Poor scalability	Python	2,260 images of Ultrasound, CT scan, and MRI

### 5.2. Generative adversarial network techniques for medical image analysis

The importance of GAN methods in medical image analysis using deep learning algorithms lies in their ability to generate realistic synthetic images, augment datasets, and improve the accuracy and effectiveness of diagnosis and analysis for various medical conditions. By the same token, in Vaccari et al. (41) the authors proposed a generative adversarial network (GAN) technique to address the issue of generating synthetic medical data for Internet of Medical Things (IoMT) applications. The authors detailed the application of their proposed method for generating a wide range of medical data samples encompassing both time series and non-time series data. They emphasized the advantages of employing a Generative Adversarial Network (GAN)-based approach, such as the capacity to generate realistic data capable of enhancing the performance of Internet of Medical Things (IoMT) systems. Through experiments utilizing authentic medical datasets like electrocardiogram (ECG) data and healthcare imaging data, the authors validated the efficacy of their proposed technique. The results demonstrated that their GAN-based method successfully produced synthetic medical data that closely resembled real medical data, both visually and statistically, as indicated by various metrics. The authors concluded that their proposed technique has the potential to be a valuable tool for generating synthetic medical data for use in IoMT applications.

Toward accurate prediction of patient length of stay at emergency.

As well, Kadri et al. (42) presented a framework that utilizes a deep learning model to predict the length of stay of patients at emergency departments. The proposed model employed a GAN to generate synthetic training data and address the problem of insufficient training data. The model used multiple input modalities, including demographic information, chief complaint, triage information, vital signs, and lab results, to predict the length of stay of patients. The authors demonstrated that their proposed framework surpassed multiple baseline models, showcasing its exceptional performance in accurately predicting the length of stay for patients in emergency departments. They recommended the deployment of the proposed framework in real-world settings, anticipating its potential to enhance the efficiency of emergency departments and ultimately improve patient outcomes.

Yang et al. (43) proposed a novel semi-supervised learning approach using GAN for clinical decision support in Health-IoT platform. The proposed model generated new samples from existing labeled data, creating additional labeled data for training. The GAN-based model undergoes training on a vast unlabeled dataset to generate medical images that exhibit enhanced realism for subsequent training purposes. These generated samples are then employed to fine-tune the pre-trained CNN, resulting in an improved classification accuracy. To assess the effectiveness of the proposed model, three medical datasets are utilized, and the findings demonstrate that the GAN-based semi-supervised learning approach surpasses the supervised learning approach, yielding superior accuracy and reduced loss values. The paper concludes that the proposed model presents the potential to enhance the accuracy of clinical decision support systems by generating supplementary training data. Furthermore, the proposed approach can be extended to diverse healthcare applications, including disease diagnosis and drug discovery.

Huang et al. (44) proposed a deep learning-based model, DU-GAN, for low-dose computed tomography (CT) denoising in the medical imaging field. The architecture of DU-GAN incorporates dual-domain U-Net-based discriminators and a GAN, aiming to enhance denoising performance and generate high-quality CT images. The proposed approach adopts a dual-domain architecture, effectively utilizing both the image domain and transform domain to differentiate real images from generated ones. DU-GAN is trained on a substantial dataset of CT images to grasp the noise distribution and noise from low-dose CT images. The results indicate that the DU-GAN model surpasses existing methods in terms of both quantitative and qualitative evaluation metrics. Furthermore, the proposed model exhibits robustness across various noise levels and different types of image data. The study showed the potential of the proposed approach for practical application in the clinical diagnosis and treatment of various medical conditions.

Purandhar et al. (45) proposes the use of Generative Adversarial Networks (GAN) for classifying clustered health care data. This study’s GAN classifier contains both a discriminator network and a generator network. While the discriminator tells the difference between genuine and false samples, the generator learns the underlying data distribution. Utilizing data from Electronic Health Records (EHRs), the MIMIC-III dataset was used by the scientists in their research. The outcomes show that the GAN classifier accurately and successfully categorizes the medical problems of patients. The authors also demonstrated the superiority of their GAN classifier by contrasting it with conventional machine learning techniques. The suggested GAN-based strategy shows promise for illness early detection and diagnosis, with potential for bettering healthcare outcomes and lowering costs. Table 4 discusses the GAN methods used in medical image analysis.

**Table 4:** The methods, properties, and features of GAN-medical image analysis mechanisms.

Author	Main idea	Advantage	Disadvantage	Simulation environment	Datasets
Vaccari, et al. (41)	Proposing a generative adversarial network (GAN) technique to address the issue of generating synthetic medical data for Internet of Medical Things (IoMT) applications	High accuracy High privacy	Limited accurately capturing the complexity Variability of real medical data using the GAN technique Longe training times	–	43 samples
Kadri, et al. (42)	Presenting a framework that utilizes a deep learning model to predict the length of stay of patients at emergency departments	High accuracy	Limited interpretability Large amount of data needed for training DL model	Python	44,676 patients
Yang, et al. (43)	Proposing a novel semi-supervised learning approach using GAN for clinical decision support in Health-IoT platform	High accuracy High robustness Cost-effective	Poor interpretability Poor security Poor privacy	Python	11,039 Stroke patient
Huang, et al. (44)	Proposing a deep learning-based model, DU-GAN, for low-dose CT denoising	High accuracy High robustness	Poor reliability Computational complexity	–	850 CT scans
Purandhar, et al. (45)	Proposing the use of GAN for classifying clustered health care data	High accuracy High robustness	Poor transparency Poor interpretability Poor sensitivity	–	452 instances

### 5.3. Recurrent neural network techniques for medical image analysis

Recurrent Neural Networks (RNNs) are essential in medical image analysis using deep learning algorithms due to their ability to capture temporal dependencies and contextual information. RNNs excel in tasks involving sequential or time-series data, such as analyzing medical image sequences or dynamic imaging modalities. Their capability to model long-term dependencies and utilize information from previous time steps enables the detection of patterns, disease progression prediction, and tracking tumor growth. RNN variants like LSTM and GRU further enhance their ability to capture complex temporal dynamics, making them vital in extracting meaningful insights from medical image sequences.

Sridhar et al. (46) proposed a novel approach for reducing the size of medical images while preserving their diagnostic quality. The authors introduced a two-stage framework that combines a Recurrent Neural Network (RNN) and a Genetic Particle Swarm Optimization with Weighted Vector Quantization (GenPSOWVQ). In the first stage, the RNN is employed to learn the spatial and contextual dependencies within the images, capturing important features for preserving diagnostic information. In the second stage, the GenPSOWVQ algorithm optimized the image compression process by selecting the best encoding parameters. The experimental results demonstrated the effectiveness of the proposed model in achieving significant image size reduction while maintaining high diagnostic accuracy. The combination of RNN and GenPSOWVQ enabled an efficient and reliable approach for medical image compression, which can have practical implications in storage, transmission, and analysis of large-scale medical image datasets.

Pham et al. (47) discussed the use of DL to predict healthcare trajectories from medical records. The authors argued that deep learning can be used to model the complex relationships between different medical conditions and predict how a patient’s healthcare journey might evolve over time. The study used data from electronic medical records of patients with various conditions, including diabetes, hypertension, and heart disease. The proposed DL model used a CNNs and RNNs to capture both the temporal and spatial relationships in the data. The research discovered that the deep learning model exhibited a remarkable ability to accurately forecast the future healthcare path of patients with a notable level of precision. The authors’ conclusion highlighted the potential of deep learning to transform healthcare delivery through enhanced accuracy in predictions and personalized care. Nevertheless, the authors acknowledged that the integration of deep learning in healthcare is still at an early phase, necessitating further investigation to fully unleash its potential.

Wang et al. (48) proposed a new approach for dynamic treatment recommendation using supervised reinforcement learning with RNNs. The authors aimed to address the challenge of making treatment decisions for patients with complex and dynamic health conditions by developing an algorithm that can adapt to changes in patient health over time. The proposed approach involved using an RNN to model patient health trajectories and predict the optimal treatment at each step. The training of the model involves a blend of supervised and reinforcement learning techniques, aimed at optimizing treatment decisions for long-term health benefits. The authors assessed the effectiveness of this approach using a dataset comprising actual patients with hypertension and demonstrated its superiority over conventional machine learning methods in terms of predictive accuracy. The suggested method holds promise in enhancing patient outcomes by offering personalized treatment recommendations that can adapt to variations in the patient’s health status.

Jagannatha and Yu (49) discusses the use of bidirectional recurrent neural networks (RNNs) for medical event detection in electronic health records (EHRs). Electronic Health Records (EHRs) offer valuable insights for medical research, yet analyzing them can be arduous due to the intricate nature and fluctuations in the data. To address this, the authors introduce a bidirectional RNN model capable of capturing the interdependencies in the sequential data of EHRs, encompassing both forward and backward relations. Through training on an EHR dataset and subsequent evaluation, the model’s proficiency in detecting medical events is assessed. The findings reveal that the bidirectional RNN surpasses conventional machine learning methods in terms of medical event detection. The authors also compare different variations of the model, such as using different types of RNNs and adding additional features to the input. Overall, the study demonstrates the potential of using bidirectional RNNs for medical event detection in EHRs, which could have important implications for improving healthcare outcomes and reducing costs.

Cocos et al. (50) focused on developing a deep learning model for pharmacovigilance to identify adverse drug reactions (ADRs) mentioned on social media platforms such as Twitter. In the study, Adverse Drug Reactions (ADRs) were trained and classified using two unique RNN architectures, namely Bidirectional Long-Short Term Memory (Bi-LSTM) and Gated Recurrent Unit (GRU). Various feature extraction methods were also looked at, and their individual performances were discussed. The outcomes showed that the Bi-LSTM model performed better than the GRU model, obtaining an F1-score of 0.86. A comparison of the deep learning models with conventional machine learning models was also done, confirming the higher performance of the deep learning models. The study focused on the possibilities of utilizing social media platforms for pharmacovigilance and underlined the efficiency of deep learning models in precisely detecting ADRs. Table 5 discusses the RNN methods used in medical image analysis.

**Table 5:** The methods, properties, and features of RNN-medical image analysis mechanisms.

Author	Main idea	Advantage	Disadvantage	Simulation environment	Datasets
Sridhar, et al. (46)	Proposing a novel approach for reducing the size of medical images	High accuracy High integrity	Costly Time-consuming High complexity	–	50 instances
Pham, et al. (47)	Proposing DL model used a CNNs and RNNs to capture both the temporal and spatial relationships in the data	High accuracy High reliability	Limited generalizability Poor scalability Poor interpretability	Python	7,191 patients
Wang, et al. (48)	Proposing a new approach for dynamic treatment recommendation	High adaptability High flexibility High generalizability	Poor availability High overfitting High complexity	–	43 K patients
Jagannatha and Yu (49)	Discussing the use of bidirectional recurrent neural networks (RNNs) for medical event detection	High robustness High flexibility High adaptability	Poor availability Computational complexity Poor interpretability	Lasagne	780 English EHR notes
Cocos, et al. (50)	Developing a DL model for pharmacovigilance to identify adverse drug reactions (ADRs)	High F-score High scalability High generalizability	Data limitation Noise and bias	Keras	844 tweets

### 5.4. Long short-term memory techniques for medical image analysis

The importance of Long Short-Term Memory (LSTM) method in medical image analysis using deep learning algorithms lies in its ability to capture and model sequential dependencies within the image data. Medical images often contain complex spatial and temporal patterns that require understanding of contextual information. LSTM, as a type of recurrent neural network (RNN), excels in modeling long-range dependencies and capturing temporal dynamics, making it suitable for tasks such as time series analysis, disease progression modeling, and image sequence analysis. By leveraging the memory and gating mechanisms of LSTM, it can effectively learn and retain relevant information over time, enabling more accurate and robust analysis of medical image data and contributing to improved diagnostic accuracy and personalized treatment in healthcare applications.

Butt et al. (51) presented a ML-based approach for diabetes classification and prediction. They used a dataset of 768 patients and 8 clinical features, including age, BMI, blood pressure, and glucose levels. Three different machine learning techniques–logistic regression, decision tree, and k-nearest neighbors–were applied to the preprocessed data before each of these algorithms was used. Sorting patients into the diabetic or non-diabetic category was the goal. Metrics including accuracy, precision, recall, and F1 score were used to evaluate the effectiveness of each method. In order to forecast the patients’ blood glucose levels, a deep learning system, namely a feedforward neural network, was used. A comparison between the performance of the deep learning algorithm and that of the traditional machine learning algorithms was conducted, revealing that the deep learning algorithm surpassed the other algorithms in terms of prediction accuracy. The authors concluded that their approach can be used for early diagnosis and management of diabetes in healthcare applications.

Awais et al. (52) proposed an Internet of Things (IoT) framework that utilizes Long Short-Term Memory (LSTM) based emotion detection for healthcare and distance learning during COVID-19. The proposed framework offers the ability to discern individuals’ emotions by leveraging physiological signals such as electrocardiogram (ECG), electrodermal activity (EDA), and photoplethysmogram (PPG). Collected data undergoes preprocessing and feature extraction prior to training an LSTM model. To assess its effectiveness, the framework is tested using the PhysioNet emotion database, where the results demonstrate its accurate emotion detection capabilities, reaching an accuracy level of up to 94.5%. With its potential applications in healthcare and distance learning amid the COVID-19 pandemic, the framework proves invaluable for remotely monitoring individuals’ emotional states and providing necessary support and interventions. The paper highlighted the importance of using IoT and machine learning in healthcare, and how it can help to address some of the challenges posed by the pandemic.

Nancy et al. (53) proposed an IoT-Cloud-based smart healthcare monitoring system for heart disease prediction using deep learning. The technology uses wearable sensors to gather physiological signs from patients, then delivers those signals to a cloud server for analysis. By training on a sizable dataset of ECG signals, a Convolutional Neural Network (CNN)-based deep learning model is used to predict cardiac illness. Transfer learning techniques, especially fine-tuning, are used to optimize the model. The suggested system’s exceptional accuracy in forecasting cardiac illness has been rigorously tested on a real-world dataset. Additionally, the model exhibits the capability to detect the early onset of heart disease, facilitating timely intervention and treatment. The paper concluded that the proposed system can be an effective tool for real-time heart disease monitoring and prediction, which can help improve patient outcomes and reduce healthcare costs.

Queralta et al. (54) presents an Edge-AI solution for fall detection in health monitoring using LoRa communication technology, fog computing, and LSTM recurrent neural networks. The proposed system consists of a wearable device, a LoRa gateway, and an edge server that processes and analyzes sensor data locally, reducing the dependence on cloud services and improving real-time fall detection. The system employs a MobileNetV2 convolutional neural network to extract features from accelerometer and gyroscope data, followed by an LSTM network that predicts falls. The authors evaluated the performance of the proposed system using a dataset collected from volunteers and achieved a sensitivity of 93.14% and a specificity of 98.9%. They also compared the proposed system with a cloud-based solution, showing that the proposed system had lower latency and reduced data transmission requirements. Overall, the proposed Edge-AI system can provide a low-cost and efficient solution for fall detection in health monitoring applications.

Gao et al. (55) introduced a novel approach called Fully Convolutional Structured LSTM Networks (FCSLNs) for joint 4D medical image segmentation. The proposed approach utilized the strengths of fully convolutional networks and structured LSTM networks to overcome the complexities arising from spatial and temporal dependencies in 4D medical image data. By integrating LSTM units into the convolutional layers, the FCSLNs successfully capture temporal information and propagate it throughout the spatial dimensions. Empirical findings strongly indicate the outstanding performance of the FCSLNs when compared to existing methods, achieving precise and resilient segmentation of 4D medical images. The proposed framework demonstrates significant promise in advancing medical image analysis tasks and enhancing clinical decision-making processes. Table 6 discusses the LSTM methods used in medical image analysis.

**Table 6:** The methods, properties, and features of LSTM-medical image analysis mechanisms.

Author	Main idea	Advantage	Disadvantage	Simulation environment	Datasets
Butt, et al. (51)	Presenting a ML-based approach for diabetes classification and prediction	High accuracy High precision High recall	Poor generalizability	–	768 records
Awais, et al. (52)	Proposing an Internet of Things (IoT) framework for healthcare and distance learning during COVID-19	High accuracy High reliability High robustness	Reliance on physiological signals for emotion detection High complexity Poor generalizability	Tensorflow	1,000 samples of data
Nancy, et al. (53)	Proposing an IoT-Cloud-based smart healthcare monitoring system for heart disease prediction using deep learning	High integrity High adaptability High availability High flexibility High scalability	Sensor errors Signal noise Less reliability High complexity	Tensorflow	100,000 records
Queralta, et al. (54)	Proposing an IoT-Cloud-based smart healthcare monitoring system for heart disease prediction	High accuracy High reliability Cost-effective High scalability	Poor security Poor privacy	Keras/Tensorflow	20 data points
Gao, et al. (55)	Introducing a novel approach called Fully Convolutional Structured LSTM Networks (FCSLNs) for joint 4D medical image segmentation	High accuracy High robustness	Poor generalizability	–	10 samples

### 5.5. Hybrid techniques for bio and medical informatics

Hybrid methods in medical image analysis, which combine deep learning algorithms with other techniques or data modalities, are of significant importance. Deep learning has demonstrated remarkable success in tasks like image segmentation and classification. However, it may face challenges such as limited training data or interpretability issues. By incorporating hybrid methods, researchers can overcome these limitations and achieve enhanced performance. Hybrid approaches can integrate traditional machine learning techniques, statistical models, or domain-specific knowledge to address data scarcity or improve interpretability. Additionally, combining multiple data modalities, such as medical images with textual reports or physiological signals, enables a more comprehensive understanding of the medical condition and facilitates better decision-making. Ultimately, hybrid methods in medical image analysis empower healthcare professionals with more accurate and reliable tools for diagnosis, treatment planning, and patient care. In this regard, Shahzadi et al. (56) proposed a novel cascaded framework for accurately classifying brain tumors using a combination of convolutional neural networks (CNNs) and long short-term memory (LSTM) networks. The proposed approach utilized the CNN’s capability to extract significant features from brain tumor images and the LSTM’s capacity to capture temporal dependencies present in the data. The cascaded framework comprised of two stages: firstly, a CNN was utilized to extract features from the tumor images, and subsequently, an LSTM network was employed to model the temporal information within these extracted features. The experimental findings clearly illustrate the exceptional performance of the CNN-LSTM framework when compared to other cutting-edge methods, exhibiting remarkable accuracy in the classification of brain tumors. The proposed method held promise for improving the diagnosis and treatment planning of brain tumors, ultimately benefiting patients and healthcare professionals in the field of neuro-oncology.

Also, Srikantamurthy et al. (57) proposed a hybrid approach for accurately classifying benign and malignant subtypes of breast cancer using histopathology imaging. Transfer learning was used to combine the strengths of long short-term memory (LSTM) networks with convolutional neural networks (CNNs) in a synergistic manner. The histopathological pictures were initially processed by the CNN to extract relevant characteristics, which were then sent into the LSTM network for sequential analysis and classification. By harnessing transfer learning, the model capitalized on pre-trained CNNs trained on extensive datasets, thereby facilitating efficient representation learning. The proposed hybrid approach showed promising results in accurately distinguishing between benign and malignant breast cancer subtypes, contributing to improved diagnosis and treatment decisions in breast cancer patients.

Besides, Banerjee et al. (58) presented a hybrid approach combining Convolutional Neural Networks (CNN) and Long Short-Term Memory (LSTM) for the classification of histopathological breast cancer images. Using data augmentation approaches, the classifier’s robustness is increased. ResNet50, InceptionV3, and a CNN that has been pretrained on ImageNet are used to extract deep convolutional features. An LSTM Recurrent Neural Network (RNN) is then fed these features for classification. Comparing the performance of three alternative optimizers, it is found that Adam outperforms the others without leading to model overfitting. The experimental findings showed that, for both binary and multi-class classification problems, the suggested strategy outperforms cutting-edge approaches. Furthermore, the method showed promise for application in the classification of other types of cancer and diseases, making it a versatile and potentially impactful approach.

Moreover, Nandhini Abirami et al. (59) explored the application of deep Convolutional Neural Networks (CNNs) and deep Generative Adversarial Networks (GANs) in computational visual perception-driven image analysis. To increase the precision and resilience of image analysis tasks, the authors suggested a unique framework that combines the advantages of both CNNs and GANs. The deep GAN is used to create realistic and high-quality synthetic pictures, while the deep CNN is used for feature extraction and capturing high-level visual representations. The combination of these two deep learning models made it possible to analyze images more efficiently, especially when performing tasks like object identification, picture recognition, and image synthesis. Experimental results demonstrated the superiority of the proposed framework over traditional approaches, highlighting the potential of combining deep CNNs and GANs for advanced computational visual perception in image analysis.

Additionally, Yao et al. (60) proposed a parallel structure deep neural network for breast cancer histology image classification, combining Convolutional Neural Networks (CNNs) and Recurrent Neural Networks (RNNs) with an attention mechanism. The histology pictures’ ability to extract both local and global characteristics thanks to the parallel construction improved the model’s capacity to gather pertinent data. The CNN component concentrated on obtaining spatial characteristics from picture patches, whereas the RNN component sequentially captured temporal relationships between patches. By focusing attention on key visual areas, the attention mechanism improved the model’s capacity for discrimination. The suggested method’s potential for accurate breast cancer histology picture categorization was shown by experimental findings, which showed that it performs better than baseline approaches. Table 7 discusses the hybrid methods used in medical image analysis.

**Table 7:** The methods, properties, and features of hybrid-medical image analysis mechanisms.

Author	Main idea	Advantage	Disadvantage	Simulation environment	Datasets
Shahzadi, et al. (56)	Proposing a novel cascaded framework for accurately classifying brain tumors	High accuracy	Computational complexity Poor interpretability Poor explainability	MATLAB	100 samples
Srikantamurthy, et al. (57)	Proposing a hybrid approach for accurately classifying benign and malignant subtypes of breast cancer	Training on a large-scale dataset High accuracy High robustness	Poor generalizability High complexity	Python	5,000 breast images
Banerjee, et al. (58)	Presenting a hybrid approach combining CNN and LSTM for the classification of histopathological breast cancer images	High accuracy High specificity High sensitivity High AUC	Poor generalizability	Tensorflow	828 samples
Nandhini Abirami, et al. (59)	Exploring the application of deep CNNs and deep GANs in computational visual perception-driven image analysis	High generalizability High adaptability High robustness High accuracy	Poor scalability	–	70,000 images
Yao, et al. (60)	Proposing a parallel structure deep neural network for breast cancer histology image classification	High accuracy	High complexity	–	100 images

## 6. Results and comparisons

**Figure 8:**
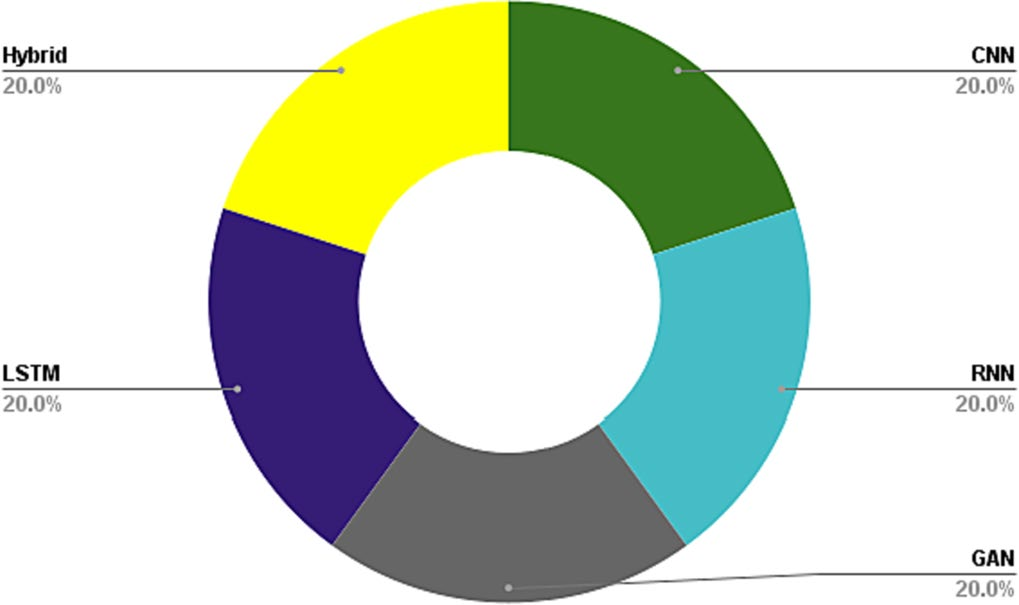
DL methods used in medical image analysis.

**Figure 9:**
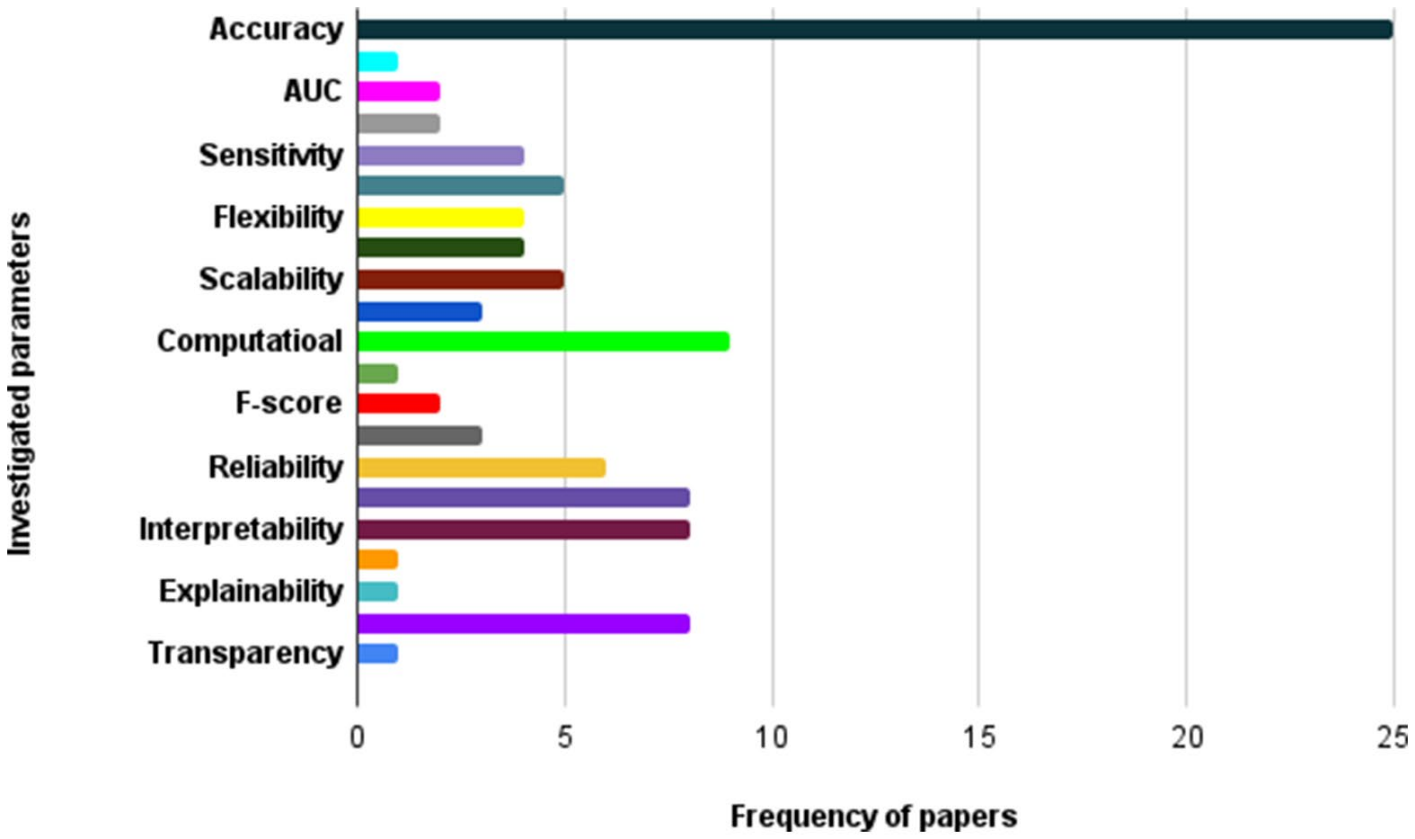
The most important parameters considered in investigated papers.

**Figure 10:**
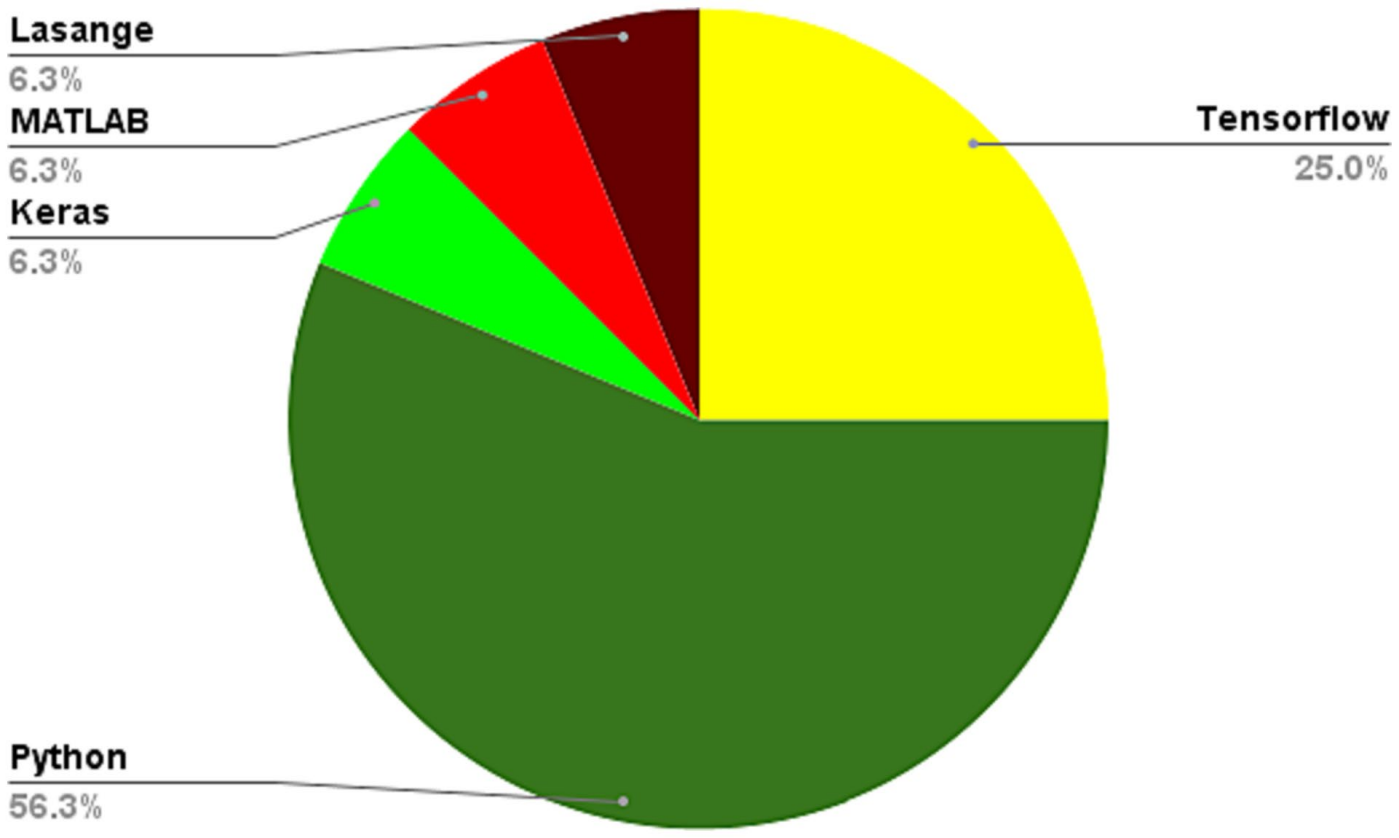
Programming languages used in learning algorithms used for medical image analysis.

The utilization of DL algorithms in medical image analysis purposes represents a pioneering stride toward the progress of medical and healthcare industries. This paper presents various innovative applications that demonstrate this paradigm, showcasing advanced knowledge in medical image analysis for motivating readers to explore innovative categories pertaining to DL algorithms in medical image analysis. The primary focus of this work is on different classifications of DL techniques utilized for DL methods in medical image analysis. Through a comprehensive analysis, it has been discovered that most DL methods in medical image analysis concentrate on advanced datasets, combined learning tasks, and annotation protocols. However, a significant limitation toward achieving the same level of functionality in medical images-DL algorithms is the inadequacy of large datasets for training, and standardized collection of data. It is crucial to ensure that diverse types of data require larger and more diverse datasets to provide reliable outcomes. Detection tasks in this field predominantly employ CNN or CNN-based techniques. In most of investigated papers the authors evaluated the topic based on several attributes, including accuracy, F-score, AUC, sensitivity, specificity, robustness, recall, adaptability, and flexibility. Sections 5.1 to 5.5 illustrate the medical image analysis-DL algorithms, where the majority of the proposed methods use both benchmark and real-time data. The DL methods used in these sections has been demonstrated in Figure 8. The systems employed various datasets in terms of numbers and diverse categories, with accuracy, computational complexity, sensitivity, specificity, robustness, generalizability, adaptability, scalability, and F-score being the primary parameters evaluated. Accuracy was the main parameter for image analysis-based systems, whereas transparency was the least applied parameter as is depicted in Figure 9. Its importance lies behind its direct impact on patient outcomes and healthcare decision-making. Medical image analysis plays a critical role in diagnosing and monitoring various diseases and conditions, and any inaccuracies or errors in the analysis can have serious consequences. High accuracy ensures that the deep learning algorithms can effectively and reliably detect abnormalities, classify different tissue types, and provide accurate predictions. This enables healthcare professionals to make well-informed decisions regarding treatment plans, surgical interventions, and disease management. Furthermore, accurate analysis helps reduce misdiagnosis rates, minimizes unnecessary procedures or tests, and improves overall patient care by enabling timely and appropriate interventions. In order to guarantee the efficiency and dependability of deep learning algorithms in medical image processing, accuracy acts as a crucial criterion. The majority of the solutions used the data normalization approach to combine photos from various sources that were of comparable size and quality. Some of the systems offered, however, did not provide the compute time since different datasets were utilized in the study. The datasets used in the study varied in terms of sample size, accessibility requirements, picture size, and classes. One of the most often employed algorithms was the RNN method, although cross-validation was seldom ever applied in most studies. Given that it is uncertain how the test results fluctuate, this might potentially reduce the outcomes’ resilience while delivering a high-functioning model. It is worth mentioning that cross-validation is crucial for evaluating the entire dataset. Multiple studies employ DL-based methodologies, and it is challenging to establish clear, robust, and resilient models. Future tasks include minimizing false-positive and false-negative rates to emphasize viral from bacterial pneumonia dependability. Associating DL methods in for developing medical image analysis represents a groundbreaking pace forward in technological development. It is worth mentioning that as is demonstrated in Figure 10, Python is the most common programming language used in this context due to several key factors. Firstly, Python offers a rich ecosystem of libraries and frameworks specifically tailored for machine learning and deep learning tasks, such as TensorFlow, PyTorch, and Keras. These libraries provide efficient and user-friendly tools for developing and deploying deep learning models. Additionally, Python’s simplicity and readability make it an accessible language for researchers, clinicians, and developers with varying levels of programming expertise. Its extensive community support and vast online resources further contribute to its popularity. Moreover, Python’s versatility allows seamless integration with other scientific computing libraries, enabling researchers to preprocess, visualize, and analyze medical image data efficiently. Its wide adoption in academia, industry, and research communities fosters collaboration and knowledge sharing among experts in the field. Overall, Python’s powerful capabilities, ease of use, and collaborative ecosystem make it the preferred choice for implementing deep learning algorithms in medical image analysis. In the domain of Medical Image Analysis using Deep Learning Algorithms, diverse methodologies are employed to extract meaningful insights from complex medical imagery. CNNs are extensively utilized for their ability to automatically identify intricate patterns and features within images. RNNs, on the other hand, are crucial when dealing with sequential medical image data, such as video sequences or time-series images, as they capture temporal dependencies. Additionally, GANs play a pivotal role, especially in tasks requiring image generation or translation. Hybrid models, which integrate different architectures like CNNs and RNNs, offer a versatile approach for handling diverse types of medical image data that may require both spatial and temporal analysis. These methodologies are implemented and simulated within specialized environments, commonly leveraging Python libraries like TensorFlow, PyTorch, and Keras, which provide comprehensive support for deep learning. GPU acceleration is often utilized to expedite model training due to the computational intensity of deep learning tasks. Furthermore, custom simulation environments may be created to mimic specific aspects of medical imaging processes. The choice of datasets is paramount; researchers may draw from open-access repositories like ImageNet for pre-training, but specialized medical imaging repositories such as TCIA or RSNA are crucial for tasks in healthcare. Additionally, custom-collected datasets tailored to specific medical image analysis tasks are often employed to ensure data relevance and quality. Data augmentation techniques, like rotation and scaling, are applied to expand datasets and mitigate limitations associated with data scarcity. These synergistic efforts in methodologies, simulation environments, and datasets are essential for the successful development and evaluation of deep learning algorithms in medical image analysis, facilitating accurate and reliable results for a wide array of healthcare applications.

### 6.1. Convolutional neural network

CNNs have been used successfully in medical image processing applications, however they also have significant drawbacks and difficulties. Due to the high expense and complexity of image collecting and annotation, it may be challenging to get the vast quantity of labeled data needed to train the network in the context of medical imaging. Additionally, the labeling procedure may add some subjectivity or inter-observer variability, which can influence the CNN models’ accuracy and dependability (61). A further issue is the possible bias of CNN models toward the distribution of training data, which might result in subpar generalization performance on fresh or untried data. This is particularly relevant in medical imaging, where the patient population may be diverse and heterogeneous, and the image acquisition conditions may vary across different imaging modalities and clinical settings. Furthermore, the interpretability of CNN models in medical imaging is still a major concern, as they typically rely on complex and opaque learned features that are difficult to interpret or explain. This limits the ability of clinicians to understand and trust the decisions made by the CNN models, and may hinder their adoption in clinical practice. Finally, CNN models are computationally intensive and require significant computational resources, which may limit their scalability and practical use in resource-constrained environments or low-resource settings (62).

The CNN method offers several benefits in the context of healthcare applications. Firstly, CNNs can automatically learn relevant features from raw input data such as medical images or physiological signals, without requiring manual feature extraction. This makes them highly effective for tasks such as image classification, object detection, and segmentation, and can lead to more accurate and efficient analyzes. Secondly, CNNs can handle large amounts of complex data and improve classification accuracy, making them well-suited for medical diagnosis and prediction (63). Additionally, CNNs can be trained on large datasets, which can help in detecting rare or complex patterns in the data that may be difficult for humans to identify. Finally, the use of deep learning algorithms such as CNNs in healthcare applications has the potential to improve patient outcomes, enable early disease detection, and reduce medical costs.

### 6.2. Recurrent neural network

Recurrent Neural Networks (RNNs) have shown great success in modeling sequential data such as time series and natural language processing tasks. However, in medical image analysis, there are some challenges and limitations when using RNNs. RNNs are mainly designed to model temporal sequences and do not have a natural way of handling spatial information in images. This can limit their ability to capture local patterns and spatial relationships between pixels in medical images. RNNs require a lot of computational power to train, especially when dealing with large medical image datasets (64). This can make it difficult to train models with high accuracy. When training deep RNN models, the gradients can either vanish or explode, making it difficult to optimize the model parameters effectively. This can lead to longer training times and lower accuracy. RNNs are prone to overfitting when the size of the training dataset is small. This can result in poor generalization performance when the model is applied to new, unseen data. Unbalanced data: In medical image analysis, the dataset may be highly unbalanced, with a small number of positive cases compared to negative cases. This can make it difficult to train an RNN model that can accurately classify the data. Researchers have created a variety of RNN-based designs, including Long Short-Term Memory (LSTM) networks and Gated Recurrent Units (GRUs), which have demonstrated promising performance in applications involving medical picture interpretation. Additionally, combining RNNs with other deep learning techniques such as CNNs can help improve performance by capturing both spatial and temporal features (65).

It’s possible that these papers faced some challenges when using the RNN method. RNNs can suffer from vanishing gradients, where the gradients used for optimization become very small and make learning slow or even impossible. This can be a challenge for RNNs when working with long sequences of data. Overfitting is a problem with RNNs, when the model gets too complicated and begins to memorize the training set rather than generalizing to new data. When working with little data, like in applications for the healthcare industry, this can be particularly difficult. RNNs may be difficult to train computationally, especially when working with big volumes of data (66). This can be a challenge when working with IoT devices that have limited computational resources. There are many different types of RNNs and architectures to choose from, each with its own strengths and weaknesses. It can be challenging to select the right architecture for a given task. Overall, while RNNs can be powerful tools for analyzing time-series data in IoT applications, they do come with some potential challenges that must be carefully considered when using them.

### 6.3. Generative adversarial network

Generative Adversarial Networks (GANs) have shown promising results in various fields, including medical image analysis. However, GANs also face some challenges and limitations, which can affect their performance in medical image analysis. Medical image datasets are often limited due to the cost and difficulty of acquiring large amounts of high-quality data. To correctly understand the underlying distribution of the data, GANs need a lot of data. Therefore, when working with tiny medical picture datasets, the performance of GANs may be constrained (67). Medical picture databases may not be evenly distributed, which means that some classifications or diseases are underrepresented. For underrepresented classes or circumstances, GANs could find it difficult to provide realistic examples. Regardless of the input, mode collapse happens when a GAN’s generator learns to produce only a small number of samples. Mode collapse in medical image processing can lead to the creation of irrational pictures or the loss of crucial data. Overfitting is a problem with GANs that happens when the model memorizes the training data rather than generalizing to. There is currently no standardization for evaluating GANs in medical image analysis. This can make it challenging to compare different GAN models and assess their performance accurately. Addressing these challenges and limitations requires careful consideration of the specific medical image analysis task, the available data, and the design of the GAN model. Moreover, a multi-disciplinary approach involving clinicians, radiologists, and computer scientists is necessary to ensure that the GAN model’s outputs are meaningful and clinically relevant (68).

### 6.4. Long short-term memory

Long Short-Term Memory (LSTM) networks are a type of recurrent neural network that has shown promising results in various applications, including medical image analysis. However, LSTMs also face some challenges and limitations, which can affect their performance in medical image analysis. LSTMs rely on a fixed-length input sequence, and the context provided by the input sequence may be limited, especially in the case of medical image analysis. For example, in a sequence of medical images, it may be challenging to capture the full context of the images in a fixed-length input sequence. LSTMs can be prone to overfitting, especially when dealing with small datasets. When the model starts to memorize the training data instead of generalizing to new, untried data, overfitting might happen. This might lead to subpar performance when the model is tested on fresh medical photos (69). LSTMs are sometimes referred to as “black box” models since it might be difficult to understand how the model generates its predictions. This can be a limitation in medical image analysis, where clinicians need to understand how the model arrived at its decision. LSTMs can be computationally expensive, especially when dealing with long input sequences or large medical image datasets. This can make it challenging to train the model on a standard computer or within a reasonable time frame. Medical image datasets can be imbalanced, meaning that certain classes or conditions are underrepresented in the dataset. LSTMs may struggle to learn the patterns of underrepresented classes or conditions. LSTMs may have limited generalizability to new medical image datasets or different medical conditions, especially if the model is trained on a specific dataset or medical condition Addressing these challenges and limitations requires careful consideration of the specific medical image analysis task, the available data, and the design of the LSTM model. Moreover, a multi-disciplinary approach involving clinicians, radiologists, and computer scientists is necessary to ensure that the LSTM model’s outputs are meaningful and clinically relevant. Additionally, techniques such as data augmentation, transfer learning, and model compression can be used to improve the performance of LSTMs in medical image analysis (70).

### 6.5. Hybrid

The reason for using hybrid methods, such as combining CNN and LSTM, is that they have complementary strengths and weaknesses. CNN is particularly good at extracting spatial features from high-dimensional data such as images, while LSTM is good at modeling temporal dependencies in sequences of data. By combining them, one can leverage the strengths of both to improve the accuracy of the prediction. Additionally, hybrid methods can be used to address challenges such as overfitting, where the model may become too specialized on the training data, and underfitting, where the model may not capture the underlying patterns in the data (71). Hybrid models can also provide a more robust approach to dealing with noisy or missing data by allowing for more complex interactions between features and time.

The use of hybrid approaches, like CNN-LSTM, in medical image analysis with deep learning algorithms, presents several challenges and limitations. Firstly, the complexity of the network architecture poses a significant hurdle in training these models. Integrating different models with diverse parameters, loss functions, and optimization algorithms can lead to suboptimal performance, potentially causing overfitting or underfitting issues, which adversely impact accuracy and generalizability (72). Secondly, a major challenge lies in obtaining a substantial amount of data to effectively train hybrid models. Medical image data is often scarce and costly to acquire, thereby restricting the capacity to train deep learning models comprehensively (73). Furthermore, medical image data’s high variability and subjectivity can compromise the training data quality and model performance. Moreover, interpreting the results generated by hybrid models can be problematic. The models’ complexity may obscure the understanding of how they arrive at predictions or classifications, limiting their practicality in clinical practice and possibly raising doubts or skepticism among medical professionals. Lastly, the computational cost associated with training and deploying hybrid models can be prohibitive (74). These models demand powerful hardware and are computationally intensive, limiting their applicability in real-world medical settings. The ability to utilize the capabilities of both models and enhance the accuracy and performance of the entire system are two advantages of utilizing hybrid approaches, such as the CNN-LSTM model. The CNN layer is utilized in the CNN-LSTM model-based COVID-19 prediction to extract spatial characteristics from the data, while the LSTM layer is used to capture temporal relationships and provide predictions based on time series data. Similar to how the CNN layer is used to extract spatial information from the EEG data in the low-invasive and low-cost BCI headband, the LSTM layer is used to collect temporal relationships and categorize the signals. When reconstructing an ECG signal using a Doppler sensor, the hybrid. Overall, the hybrid models can provide better performance and accuracy compared to using either model alone (75).

The utilization of hybrid methods, such as the CNN-LSTM model, offers various advantages, including the amalgamation of both models’ strengths to enhance the overall system’s accuracy and performance. For instance, the CNN layer is used to extract spatial characteristics from the data in the COVID-19 prediction using the CNN-LSTM model, while the LSTM layer collects temporal relationships and makes predictions based on the time series data. Similar to how the CNN layer gets spatial information from the EEG data in the instance of EEG detection using a low-invasive and affordable BCI headband, the LSTM layer captures temporal relationships and categorizes the signals (76). The hybrid model makes use of the CNN layer to extract high-level features from the Doppler signal in the context of reconstructing an ECG signal using a Doppler sensor, and the LSTM layer makes use of the derived features to help reconstruct the ECG signal. In summary, employing hybrid models can yield superior performance and accuracy compared to using either model individually. This approach enables the combination of spatial and temporal information, harnessing the strengths of both CNN and LSTM models to enhance various applications such as COVID-19 prediction, EEG detection, and ECG signal reconstruction.

### 6.6. Prevalent evaluation criteria

Due to its capacity to increase the precision and efficacy of medical diagnosis and therapy, deep learning algorithms for medical image analysis have grown in popularity in recent years. In evaluating the performance of deep learning algorithms in medical image analysis, there are several prevalent evaluation criteria, which are described below (13).

#### 6.6.1. Accuracy

Accuracy is the most commonly used metric for evaluating the performance of deep learning algorithms in medical image analysis. It measures the percentage of correctly classified images or regions of interest (ROIs) in medical images.


(1) $$\mathrm{Accuracy}={\frac{STN+STP}{STP+STN+SFN+SFP}}^{*}\;100$$

#### 6.6.2. Sensitivity and specificity

Sensitivity measures the proportion of true positive results, which are the number of positive cases that are correctly identified by the algorithm. Specificity measures the proportion of true negative results, which are the number of negative cases that are correctly identified by the algorithm. Both metrics are used to evaluate the diagnostic performance of deep learning algorithms in medical image analysis.


(2) $$\mathrm{Sensitivity}=\frac{TP}{TP+FN}$$

(3) $$\mathrm{Specificity}=\frac{TN}{STN+FP}$$

#### 6.6.3. Precision and recall

Precision measures the proportion of true positive results among all the positive cases identified by the algorithm. Recall measures the proportion of true positive results among all the positive cases in the ground truth data. Both metrics are used to evaluate the performance of deep learning algorithms in medical image analysis, particularly in binary classification tasks.

(4) $$\mathrm{Precision}={\frac{STP}{STP+SFP}}^{*}\;100$$

(5) $$\mathrm{Recall}=\;{\frac{STP}{STP+SFN}}^{*}\;100$$

#### 6.6.4. F1-score

The F1-score is a metric that combines precision and recall into a single score. It is often used to evaluate the performance of deep learning algorithms in medical image analysis, particularly in binary classification tasks.


(6) $$F‐\mathrm{score}=2^{*}\;\frac{{\mathrm{Precision}}^{*}\;\mathrm{Recall}}{\mathrm{Precision}+\mathrm{Recall}}$$

#### 6.6.5. Hausdorff distance

The Hausdorff distance is a metric that measures the maximum distance between the boundaries of two sets of ROIs in medical images. It is often used to evaluate the segmentation accuracy of deep learning algorithms in medical image analysis.

In general, the unique task and setting of the medical image analysis determine the selection of assessment criteria. In order to evaluate the outcomes of deep learning algorithms in the context of clinical practice, it is crucial to choose appropriate assessment criteria that are pertinent to the therapeutic demands.

### 6.7. Challenges of the DL applications in medical image analysis

The lack of high-quality annotated data is one of the greatest problems with deep learning (DL) algorithms used for medical image analysis. For DL models to perform well and generalize, they need a lot of labeled data. But getting high-quality annotations for medical photos is challenging for a number of reasons: restricted accessibility: Because it is expensive and time-consuming to capture and annotate medical pictures, the amount of data from annotated images is constrained (76). Additionally, the process of annotating calls for medical professionals with particular training and understanding, who are not always available. Due to changes in patient anatomy, imaging modality, and disease pathology, medical pictures are complicated and extremely varied. Annotating medical images requires a high degree of accuracy and consistency, which can be challenging for complex and heterogeneous medical conditions. Privacy and ethical issues: The annotation process has the potential to make medical photographs containing sensitive patient data vulnerable to abuse or unauthorized access. Medical image analysis has a significant difficulty in protecting patient privacy and confidentiality while preserving the caliber of annotated data. Annotating medical pictures requires making subjective assessments, which might result in bias and variability in the annotations. These variables may have an impact on the effectiveness and generalizability of DL models, especially when the annotations are inconsistent among datasets or annotators (77). To address the challenge of limited availability of high-quality annotated data, several approaches have been proposed, including:

- Transfer learning: To enhance the performance of DL models on smaller datasets, transfer learning uses pre-trained models that have been learned on big datasets. By using this method, the volume of annotated data needed to train DL models may be decreased, and the generalizability of the models can be increased.
- Data augmentation: By applying modifications to already-existing, annotated data, data augmentation includes creating synthetic data. The diversity and quantity of annotated data available for DL model training may be increased using this method, and it can also raise the models’ resistance to fluctuations in medical pictures.
- Active learning: Active learning involves selecting the most informative and uncertain samples for annotation, rather than annotating all the data. This approach can reduce the annotation workload and improve the efficiency of DL model training.
- Collaborative annotation: Collaborative annotation involves engaging medical experts, patients, and other stakeholders in the annotation process to ensure the accuracy, consistency, and relevance of annotations to clinical needs and values.

Overall, addressing the challenge of limited availability of high-quality annotated data in medical image analysis requires a combination of technical, ethical, and social solutions that can improve the quality, quantity, and diversity of annotated data while ensuring patient privacy and ethical standards.

Deep learning algorithms for medical image analysis have a significant problem in terms of data quality. The model’s performance may be considerably impacted by the caliber of the data utilized to train the deep learning algorithms (78). Obtaining medical pictures may be difficult, and their quality can vary based on a number of variables, such as the image capture equipment used, the image resolution, noise, artifacts, and the imaging technique. Furthermore, the annotations or labels used for training can also impact the quality of the data. Annotations may not always be accurate, and they may suffer from inter-and intra-observer variability, which can lead to biased models or models with poor generalization performance. To overcome the challenge of data quality, researchers need to establish robust quality control procedures for both image acquisition and annotation. Additionally, they need to develop algorithms that can handle noisy or low-quality data and improve the accuracy of annotations. Finally, they need to develop methods to evaluate the quality of the data used to train the deep learning models (79).

Interpretability poses a significant challenge in medical image analysis when employing deep learning algorithms, primarily due to the conventional black-box nature of these models, which makes it arduous to comprehend the reasoning behind their predictions. This lack of interpretability hinders clinical acceptance, as healthcare professionals necessitate understanding and trust in a model’s decision-making process to utilize it effectively. Moreover, interpretability plays a vital role in identifying and mitigating biases within the data and model, ensuring that decisions are not influenced by irrelevant or discriminatory features. Various approaches have been developed to enhance the interpretability of deep learning models for medical image analysis (80). These approaches include visualization techniques, saliency maps, and model explanations. Nonetheless, achieving complete interpretability remains a challenge in this field as it necessitates a trade-off between model performance and interpretability. Striking the right balance between these factors remains an ongoing endeavor. Transferability refers to the ability of a deep learning model trained on a particular dataset to generalize and perform well on new datasets that have different characteristics. In the context of medical image analysis, transferability is a significant challenge due to the diversity of medical imaging data, such as variations in image quality, imaging protocols, and imaging modalities. Deep learning models that are trained on a specific dataset may not perform well on different datasets that have variations in data quality and imaging characteristics. This can be problematic when developing deep learning models for medical image analysis because it is often not feasible to train a new model for every new dataset. To address this challenge, researchers have explored techniques such as transfer learning and domain adaptation. Transfer learning involves using a pre-trained model on a different but related dataset to initialize the model weights for the new dataset, which can improve performance and reduce the amount of training required. Domain adaptation involves modifying the model to account for the differences between the source and target domains, such as differences in imaging protocols or modalities (81). However, the challenge of transferability remains a significant issue in medical image analysis, and there is ongoing research to develop more robust and transferable deep learning models for this application.

In deep learning-based medical image analysis, overfitting is a frequent problem when a model gets overly complicated and fits the training data too closely, leading to poor generalization to new, unforeseen data. Numerous factors, including the inclusion of noise in the training data, an unbalanced class distribution, or a lack of training data, can lead to overfitting (64). The latter is a prevalent problem in medical imaging since the dataset size is constrained by the absence of annotated data. Overfitting can provide erroneous positive or negative findings because it can produce high accuracy on training data but poor performance on validation or testing data. To avoid overfitting in deep learning, several strategies may be used, including regularization, early halting, and data augmentation. In medical image analysis, ensuring the quality of data and increasing the size of the dataset are essential to prevent overfitting.

Clinical adoption refers to the process of integrating new technologies or methodologies into clinical practice. In the context of medical image analysis using deep learning algorithms, clinical adoption is a challenge because it requires a significant change in how physicians and healthcare providers diagnose and treat patients (82). Clinical adoption involves not only technical considerations such as integrating the algorithms into existing systems and workflows, but also ethical, legal, and regulatory considerations, as well as training healthcare providers to use the new technology effectively and safely. One of the key challenges of clinical adoption is ensuring that the deep learning algorithms are accurate and reliable enough to be used in clinical decision-making. This requires rigorous validation and testing of the algorithms, as well as addressing concerns around the interpretability and generalizability of the results. Additionally, healthcare providers and patients may have concerns about the use of these algorithms in making medical decisions, particularly if the algorithms are seen as replacing or minimizing the role of the human clinician. Another challenge of clinical adoption is the need for regulatory approval, particularly in cases where the algorithms are used to support diagnosis or treatment decisions. Regulatory bodies such as the FDA may require clinical trials to demonstrate the safety and effectiveness of the algorithms before they can be used in clinical practice. The adoption of these technologies may be slowed down by this procedure since it can be time-consuming and expensive. Overall, clinical adoption is an important challenge to consider in the development and deployment of medical image analysis using deep learning algorithms, as it affects the ultimate impact of these technologies on patient care (83).

### 6.8. Dataset in medical image analysis using ML algorithms

In medical image analysis, a dataset is a collection of medical images that are used to train machine learning algorithms to detect and classify abnormalities or diseases. The dataset could be obtained from various sources such as clinical trials, imaging studies, or public repositories (84). The dataset’s data quality and size have a significant impact on how well the machine learning algorithm performs. Therefore, a dataset should be diverse and representative of the population under study to ensure the accuracy and generalizability of the algorithm. In addition, datasets may require pre-processing, such as normalization or augmentation, to address issues such as data imbalance, low contrast, or artifacts. A fundamental issue in the field of medical image analysis is still finding and using big, carefully managed medical picture databases. However, efforts are underway to improve the quality and availability of medical image datasets for researchers to advance the development of ML algorithms for medical diagnosis and treatment. In medical image analysis using machine learning (ML) algorithms, a dataset is a collection of images that are used to train and test ML models. Any ML project must include a dataset since the dataset’s size and quality directly affect how well the model performs. Obtaining and annotating medical photos from a variety of sources, including hospitals, clinics, and research organizations, is a standard step in the process of producing a dataset (85). To specify the areas of interest or characteristics that the ML model needs to learn, the pictures must be tagged. These labels could provide details about the disease shown in the picture, the anatomy of the area being imaged, or other pertinent facts. The training set and the test set are formed once the dataset is first established. The ML model is trained using the training set, and tested using the test set. As such, there is ongoing research in the field of medical image analysis aimed at improving dataset quality and size, as well as developing better methods for acquiring and labeling medical images (74, 86).

### 6.9. Security issues, challenges, risks, IoT and blockchain usage

Medical image analysis using deep learning algorithms raises several security issues, particularly with regard to patient privacy and data protection. The medical images used for training the deep learning models may contain sensitive information, such as personally identifiable information (PII), health records, and demographic information, which must be kept confidential and secure. One of the main security issues is the risk of data breaches, which can occur during the data collection, storage, and transmission stages. Hackers or unauthorized personnel can intercept the data during transmission, gain access to the storage systems, or exploit vulnerabilities in the software or hardware infrastructure used to process the data (13). To mitigate this risk, various security measures must be put in place, such as encryption, access controls, and monitoring tools (87). Another security issue is the possibility of malicious attacks on the deep learning models themselves. Attackers can attempt to manipulate the models’ outputs by feeding them with malicious inputs, exploiting vulnerabilities in the models’ architecture or implementation, or using adversarial attacks to deceive the models into making wrong predictions. This can have serious consequences for patient diagnosis and treatment, and thus, it is critical to design and implement secure deep learning models. In summary, security is a critical concern in medical image analysis using deep learning algorithms, and it is essential to adopt appropriate security measures to protect the confidentiality, integrity, and availability of medical data and deep learning models.

There are several risks associated with medical image analysis using deep learning algorithms. Some of the key risks include:

- Inaccuracy: Deep learning algorithms may sometimes provide inaccurate results, which can lead to incorrect diagnoses or treatment decisions.
- Bias: Deep learning algorithms may exhibit bias in their decision-making processes, leading to unfair or inaccurate results for certain groups of patients.
- Privacy concerns: Medical images often contain sensitive information about patients, and there is a risk that this data could be exposed or compromised during the analysis process.
- Cybersecurity risks: As with any technology that is connected to the internet or other networks, there is a risk of cyberattacks that could compromise the security of medical images and patient data.
- Lack of transparency: Deep learning algorithms can be difficult to interpret, and it may be challenging to understand how they arrive at their conclusions. This lack of transparency can make it difficult to trust the results of the analysis.

Overall, it is important to carefully consider these risks and take steps to mitigate them when using deep learning algorithms for medical image analysis. This includes implementing strong cybersecurity measures, ensuring data privacy and confidentiality, and thoroughly validating the accuracy and fairness of the algorithms.

The term “Internet of Things” (IoT) describes how physical “things” are linked to the internet so they can trade and gather data. IoT may be used to link medical imaging devices and enable real-time data collecting and analysis in the field of medical image analysis. For instance, a network may be used to connect medical imaging equipment like CT scanners, MRIs, and ultrasounds, which can then transfer data to a cloud-based system for analysis (88). This can facilitate remote consultations and diagnostics and speed up the examination of medical images. IoT can also make it possible to combine different medical tools and data sources, leading to more thorough and individualized patient treatment. However, the use of IoT in medical image analysis also raises security and privacy concerns, as sensitive patient data is transmitted and stored on a network that can be vulnerable to cyber-attacks.

## 7. Open issues

There are several open issues related to medical image analysis using deep learning algorithms. These include:

### 7.1. Data privacy

Data privacy is a major concern in medical image analysis using deep learning algorithms. Medical images contain sensitive patient information that must be kept confidential and secure. In order to secure patient data from illegal access, usage, or disclosure, any algorithm or system used for medical image analysis must follow this rule. This can be particularly difficult since medical image analysis sometimes involves enormous volumes of data, which raises the possibility of data breaches or unwanted access. The need to strike a balance between the demands of data access and patient privacy protection is one of the primary issues with data privacy in medical image analysis. Many medical image analysis algorithms rely on large datasets to achieve high levels of accuracy and performance, which may require sharing data between multiple parties (89). This can be particularly challenging when dealing with sensitive patient information, as there is a risk of data leakage or misuse. Several methods may be utilized to protect data privacy in medical image analysis in order to deal with these issues. These include rules and processes to guarantee that data is accessed and used only for legal purposes, data anonymization, encryption, and access restrictions. Additionally, to guarantee that patient data is safeguarded and handled properly, healthcare companies must ensure that they adhere to pertinent data privacy laws, such as HIPAA in the United States or GDPR in the European Union.

### 7.2. Data bias

When employing deep learning algorithms to analyze medical images, data bias is a serious open problem. It alludes to the fact that the data used to train the deep learning models contains systematic flaws (90). These blunders may result from variables including the choice of training data, how the data is labeled, and how representative the data are of the population of interest. Data bias can result in the creation of models that underperform on particular segments of the population, such as members of underrepresented groups or those who suffer from unusual medical diseases. This can have serious implications for the accuracy and fairness of medical image analysis systems, as well as for the potential harm caused to patients if the models are used in clinical decision-making. Addressing data bias requires careful consideration of the data sources, data labeling, and model training strategies to ensure that the models are representative and unbiased (91).

### 7.3. Limited availability of annotated data

Deep learning algorithms in medical image analysis need a lot of annotated data to be taught properly. Annotated data refers to medical images that have been labeled by experts to indicate the location and type of abnormalities, such as tumors, lesions, or other pathologies. However, obtaining annotated medical image datasets is particularly challenging due to several factors. First off, annotating medical photos takes time and requires in-depth understanding. Only experienced radiologists or clinicians can accurately identify and label abnormalities in medical images, which can limit the availability of annotated data. Secondly, there are privacy concerns associated with medical image data. Patient privacy is a critical concern in healthcare, and medical image data is considered particularly sensitive (92). As a result, obtaining large-scale annotated medical image datasets for deep learning is challenging due to privacy concerns and the need to comply with regulations such as HIPAA. Thirdly, the diversity of medical image data can also pose a challenge. Medical images can vary widely in terms of modality, acquisition protocols, and image quality, making it difficult to create large, diverse datasets for deep learning. Deep learning algorithms for medical image analysis may be limited in their ability to develop and be validated as a result of the difficulties in getting datasets of annotated medical images. In order to decrease the volume of labeled data needed for training, researchers have tackled this issue by adopting methods including transfer learning, data augmentation, and semi-supervised learning (93). However, these techniques may not be sufficient in all cases, and there is a need for more annotated medical image datasets to be made available to researchers to advance the field of medical image analysis using deep learning.

### 7.4. Interpretability and transparency

When employing deep learning algorithms for medical picture analysis, interpretability and transparency are crucial concerns. Deep learning models are sometimes referred to as “black boxes” because they can be tricky to read, making it difficult to comprehend how they made judgments. In medical image analysis, interpretability is essential for clinicians to understand and trust the algorithms, as well as to identify potential errors or biases. Interpretability refers to the ability to understand the reasoning behind a model’s decision-making process. Convolutional neural networks (CNNs), one type of deep learning model, can include millions of parameters that interact in intricate ways. This complexity can make it difficult to understand how the model arrived at a particular decision, especially for clinicians who may not have experience with deep learning. Transparency refers to the ability to see inside the model and understand how it works (94). In other words, transparency means that the model’s decision-making process is clear and understandable, and can be validated and audited. Transparency is essential for ensuring that the model is working correctly and not introducing errors or biases. In medical image analysis, interpretability and transparency are critical because clinicians need to understand how the algorithm arrived at its decisions. This understanding can help clinicians identify errors or biases and ensure that the algorithm is making decisions that are consistent with clinical practice. To increase the interpretability and transparency of deep learning models in medical image analysis, several techniques have been developed. For instance, heatmaps that display which areas of an image the model is utilizing to make judgments may be produced using visualization approaches. Additionally, attention mechanisms can be used to highlight important features in an image and explain the model’s decision-making process. Other techniques include using explainable AI (XAI) methods and incorporating domain knowledge into the models. While these techniques have shown promise, there is still a need for more transparent and interpretable deep learning models in medical image analysis to improve their utility in clinical practice.

### 7.5. Generalizability

A significant unresolved problem in deep learning-based medical picture analysis is generalizability. The capacity of a model to function effectively on data that differs from the data it was trained on is referred to as generalizability. In other words, a trained model should be able to generalize to other datasets and still perform well. In medical image analysis, generalizability is critical because it ensures that the deep learning algorithms can be used on new patient populations or in different clinical settings. However, deep learning models can be prone to overfitting, which occurs when a model performs well on the data it was trained on but performs poorly on new data. This can be particularly problematic in medical image analysis, where a model that overfits can lead to inaccurate or inconsistent diagnoses. The generalizability of deep learning models for medical image processing might vary depending on a number of variables. For instance, a model’s capacity to generalize can be significantly impacted by the variety of the dataset used to train it (95). The model might not be able to identify anomalies that it has never seen before if the training dataset is not sufficiently varied. Another factor that can affect generalizability is the performance of the model on different types of medical images. For example, a model that is trained on CT scans may not perform well on MRI scans because the image modality and acquisition protocols are different. Researchers are examining methods including transfer learning, data augmentation, and domain adaptation to increase the generalizability of deep learning models in medical picture analysis. Transfer learning entails fine-tuning a pre-trained model using a fresh dataset as a starting point. Data augmentation entails using transformations like rotations and translations to artificially expand the size and variety of the training dataset. The process of domain adaptation is modifying a model that has been trained on one dataset to function on another dataset with different properties. The generalizability of deep learning models in medical image processing has to be improved in order to assure their safe and efficient application in clinical practice, even if these approaches have showed promise (96).

### 7.6. Validation and regulatory approval

Validation and regulatory approval are important open issues in medical image analysis using deep learning algorithms. Validation refers to the process of verifying that a model is accurate and reliable. Regulatory approval refers to the process of obtaining approval from regulatory bodies, such as the FDA in the US, before a model can be used in clinical practice. Validation is critical in medical image analysis because inaccurate or unreliable models can lead to incorrect diagnoses and treatment decisions. Validation involves testing the model on a separate dataset that was not used for training and evaluating its performance on a range of metrics. Validation can also involve comparing the performance of the model to that of human experts. Regulatory approval is important in medical image analysis to ensure that the models are safe and effective for use in clinical practice. Regulatory bodies require evidence of the model’s safety, efficacy, and performance before approving it for use. This evidence can include clinical trials, real-world data studies, and other forms of validation. There are several challenges associated with validation and regulatory approval of deep learning models in medical image analysis. One challenge is the lack of standardized validation protocols, which can make it difficult to compare the performance of different models (97). Another challenge is the lack of interpretability and transparency of deep learning models, which can make it difficult to validate their performance and ensure their safety and efficacy. Researchers and regulatory organizations are collaborating to provide standardized validation processes and criteria for regulatory approval of deep learning models in medical image analysis in order to overcome these issues. For instance, the FDA has published guidelines for the creation and approval of medical devices based on machine learning and artificial intelligence (AI/ML). These guidelines provide recommendations for the design and validation of AI/ML-based medical devices, including those used for medical image analysis. While these efforts are promising, there is still a need for further research and collaboration between researchers and regulatory bodies to ensure the safe and effective use of deep learning models in medical image analysis (98).

### 7.7. Ethical and legal considerations

Deep learning algorithms for medical image processing raise a number of significant outstanding questions about moral and legal dilemmas. These factors concern the use of patient data in research, the possibility of algorithmic biases, and the duty of researchers and healthcare professionals to guarantee the ethical and safe application of these technologies. Use of patient data in research is one ethical issue. Large volumes of patient data are needed for medical image analysis, and the use of this data raises questions concerning patient privacy and permission. Patients’ privacy must be maintained, and researchers and healthcare professionals must make sure that patient data is utilized responsibly (99). The possibility for prejudice in algorithms is another ethical issue. Deep learning algorithms may be taught on skewed datasets, which might cause the model’s outputs to become biased. Biases can result in incorrect diagnosis and treatment choices in medical image analysis, which can have catastrophic repercussions. Researchers must take action to address any potential biases in their datasets and algorithms. Deep learning algorithms for medical image interpretation raise legal questions around intellectual property, liability, and compliance with regulations. Concerns exist around the possibility of unwanted access to patient data as well as the requirement to uphold data protection regulations in order to preserve patient privacy. To address these ethical and legal considerations, researchers and healthcare providers must ensure that they are following best practices for data privacy and security, obtaining informed consent from patients, and working to mitigate potential biases in their algorithms. It is also important to engage with stakeholders, including patients, regulatory bodies, and legal experts, to ensure that the development and use of these technologies is safe, ethical, and compliant with relevant laws and regulations (100).

### 7.8. Future works

Future research in the fast-developing field of medical image analysis utilizing deep learning algorithms has a lot of potential to increase the precision and effectiveness of medical diagnosis and therapy. Some of these areas include:

#### 7.8.1. Multi-modal image analysis

Future research in medical image analysis utilizing deep learning algorithms will focus on multi-modal picture analysis. Utilizing a variety of imaging modalities, including MRI, CT, PET, ultrasound, and optical imaging, allows for a more thorough understanding of a patient’s anatomy and disease (101). This strategy can aid in enhancing diagnostic precision and lowering the possibility of missing or incorrect diagnoses. Multi-modal picture data may be used to train deep learning algorithms for a range of tasks, including segmentation, registration, classification, and prediction. An algorithm built on MRI and PET data, for instance, might be used to identify areas of the brain afflicted by Alzheimer’s disease. Similarly, a deep learning algorithm could be trained on ultrasound and CT data to identify tumors in the liver. Multi-modal image analysis poses several challenges for deep learning algorithms. For example, different imaging modalities have different resolution, noise, and contrast characteristics, which can affect the performance of the algorithm. Additionally, multi-modal data can be more complex and difficult to interpret than single-modality data, requiring more advanced algorithms and computational resources (102). To address these challenges, researchers are developing new deep learning models and algorithms that can integrate and analyze data from multiple modalities. For example, multi-modal fusion networks can be used to combine information from different imaging modalities, while attention mechanisms can be used to focus the algorithm’s attention on relevant features in each modality. Overall, multi-modal image analysis holds promise for improving the accuracy and efficiency of medical diagnosis and treatment using deep learning algorithms. As these technologies continue to evolve, it will be important to ensure that they are being used safely, ethically, and in accordance with relevant laws and regulations.

#### 7.8.2. Explainable AI

Future research in deep learning algorithms for medical image analysis will focus on explainable AI (XAI). XAI is the capacity of an AI system to explain its decision-making process in a way that is intelligible to a human (103). XAI can assist to increase confidence in deep learning algorithms when employed in the context of medical image analysis, guarantee that they are utilized safely and morally, and allow clinicians to base their judgments more intelligently on the results of these algorithms. XAI in medical image analysis involves developing algorithms that can not only make accurate predictions or segmentations but also provide clear and interpretable reasons for their decisions. This can be particularly important in cases where the AI system’s output contradicts or differs from the clinician’s assessment or prior knowledge. One approach to XAI in medical image analysis is to develop visual explanations or heatmaps that highlight the regions of an image that were most important in the algorithm’s decision-making process. These explanations can help to identify regions of interest, detect subtle abnormalities, and provide insight into the algorithm’s thought process (104). Another approach to XAI in medical image analysis is to incorporate external knowledge or prior information into the algorithm’s decision-making process. For example, an algorithm that analyzes brain MRIs could be designed to incorporate known patterns of disease progression or anatomical landmarks. Overall, XAI holds promise for improving the transparency, interpretability, and trustworthiness of deep learning algorithms in medical image analysis. As these technologies continue to evolve, it will be important to ensure that they are being used safely, ethically, and in accordance with relevant laws and regulations (105).

#### 7.8.3. Transfer learning

Future research in the field of deep learning-based medical image processing will focus on transfer learning. Transfer learning is the process of using previously trained deep learning models to enhance a model’s performance on a new task or dataset. Transfer learning can be particularly helpful in the interpretation of medical images as it can eliminate the requirement for significant volumes of labeled data, which can be challenging and time-consuming to gather. Researchers can use pre-trained models that have already been trained on huge datasets to increase the precision and effectiveness of their own models by taking advantage of the information and representations acquired by these models. Since transfer learning can do away with the need for large amounts of labeled data, which can be difficult and time-consuming to collect, it can be very useful in the interpretation of medical pictures. By utilizing the knowledge and representations amassed by pre-trained models that have previously been trained on massive datasets, researchers may utilize them to improve the accuracy and efficacy of their own models (106). The pre-trained model could be a useful place to start for the medical image analysis problem since it enables the model to learn from less data and might lessen the possibility of overfitting. Additionally, transfer learning may increase the generalizability of deep learning models used for medical picture interpretation. Medical image analysis models may be able to develop more reliable and generalizable representations of medical pictures that are relevant to a wider range of tasks and datasets by making use of pre-trained models that have learnt representations of real images. Transfer learning has the potential to enhance the effectiveness, precision, and generalizability of deep learning models used for medical image interpretation. As these technologies continue to evolve, it will be important to ensure that they are being used safely, ethically, and in accordance with relevant laws and regulations.

#### 7.8.4. Federated learning

Future research in deep learning algorithms for medical image analysis will focus on federated learning. Without the need to move the data to a central server, federated learning refers to the training of machine learning models on data that is dispersed among several devices or institutions. Federated learning can be especially helpful in the context of medical image analysis since it permits the exchange of information and expertise between institutions while safeguarding the confidentiality and security of sensitive patient data (107). In situations where patient data is subject to strong privacy laws, such as the Health Insurance Portability and Accountability Act (HIPAA) in the United States, this can be particularly crucial. Federated learning works by training a central machine learning model on a set of initial weights, which are then sent to each of the participating devices or institutions. Each device or institution then trains the model on their own local data, using the initial weights as a starting point. The updated weights from each device or institution are then sent back to the central server, where they are aggregated to update the central model. This process is repeated iteratively until the model converges. By training models using federated learning, medical institutions can leverage the collective knowledge and expertise of multiple institutions, improving the accuracy and generalizability of the models. Additionally, the confidentiality and privacy of patient data are preserved because the data stays on local devices or organizations. Overall, federated learning shows potential for enhancing deep learning models’ generalizability, speed, and privacy in the context of medical picture analysis (108). As these technologies continue to evolve, it will be important to ensure that they are being used safely, ethically, and in accordance with relevant laws and regulations.

#### 7.8.5. Integration with electronic health records (EHRs)

Future development in deep learning algorithms for medical image analysis will focus on integration with electronic health records (EHRs). EHRs contain a wealth of clinical information, including patient demographics, medical history, laboratory results, and imaging studies. Researchers and clinicians may be able to increase the precision and effectiveness of medical image analysis by merging deep learning algorithms with EHRs. One potential application of this integration is to improve the interpretation of medical images by incorporating patient-specific information from EHRs. For example, deep learning algorithms could be trained to predict the likelihood of certain diseases or conditions based on a patient’s clinical history, laboratory results, and imaging studies. This may decrease the need for invasive or pricey diagnostic procedures and increase the accuracy of medical picture interpretation. Using deep learning algorithms to automatically extract data from medical photos and incorporate it into EHRs is a further possible use (109). For example, deep learning algorithms could be trained to automatically segment and measure lesions or tumors in medical images and record this information in the patient’s EHR. This may decrease the need for invasive or pricey diagnostic procedures and increase the accuracy of medical picture interpretation. Using deep learning algorithms to automatically extract data from medical photos and incorporate it into EHRs is a further possible use. This may lessen the strain on physicians and increase the effectiveness and precision of clinical decision-making. Overall, deep learning algorithm integration with EHRs shows potential for enhancing the precision, efficacy, and efficiency of medical picture processing. It will be crucial to make sure that these technologies are utilized safely, morally, and in line with all applicable laws and regulations regarding patient privacy and data security as they continue to advance (110).

#### 7.8.6. Few-shots learning

Future research in Medical Image Analysis using DL algorithms should delve into the realm of Few-shot Learning. This approach holds great potential for scenarios where labeled data is limited or difficult to obtain, which is often the case in medical imaging (111). Investigating techniques that enable models to learn from a small set of annotated examples, and potentially even adapt to new, unseen classes, will be instrumental. Meta-learning algorithms, which aim to train models to quickly adapt to new tasks with minimal data, could be explored for their applicability in medical image analysis. Additionally, methods for data augmentation and synthesis specifically tailored for few-shot scenarios could be developed. By advancing Few-shot Learning in the context of medical imaging, we can significantly broaden the scope of applications, improve the accessibility of AI-driven healthcare solutions, and ultimately enhance the quality of patient care (112).

## 8. Conclusion and limitation

In recent years, there has been significant progress in medical image analysis using deep learning algorithms, with numerous studies highlighting the effectiveness of DL in various areas like cell, bone, tissue, tumor, vessel, and lesion segmentation. However, as the field continues to evolve, further research is essential to explore new techniques and methodologies that can improve the performance and robustness of DL algorithms in image analysis. Comprehensive evaluations of DL algorithms in real-world scenarios are needed, along with the development of scalable and robust systems for healthcare settings. Continuing research in this area is imperative to fully utilize the potential of DL in medical image segmentation and enhance healthcare outcomes. This article presents a systematic review of DL-based methods for image analysis, discussing advantages, disadvantages, and the strategy employed. The evaluation of DL-image analysis platforms and tools is also covered. Most papers are assessed based on qualitative features, but some important aspects like security and convergence time are overlooked. Various programming languages are used to evaluate the proposed methods. The investigation aims to provide valuable guidance for future research on DL application in medical and healthcare image analysis. However, the study encountered constraints, including limited access to non-English papers and a scarcity of high-quality research focusing on this topic. The heterogeneity in methodologies, datasets, and evaluation metrics used in the studies presents challenges in drawing conclusive insights and performing quantitative meta-analysis. Additionally, the rapidly evolving nature of DL techniques and the emergence of new algorithms may necessitate frequent updates to remain current. Despite these limitations, DL has proven to be a game-changing approach for addressing complex problems, and the study’s results are expected to advance DL approaches in real-world applications.

## Data availability statement

The original contributions presented in the study are included in the article/supplementary material, further inquiries can be directed to the corresponding author.

## Author contributions

ML: Investigation, Writing – original draft. YJ: Investigation, Writing – review & editing. YZ: Investigation, Supervision, Writing – original draft. HZ: Investigation, Writing – original draft.
